# Open-[60]fullerene–aniline conjugates with near-infrared absorption†

**DOI:** 10.1039/d3ra02113k

**Published:** 2023-05-12

**Authors:** Shumpei Sadai, Yoshifumi Hashikawa, Yasujiro Murata

**Affiliations:** a Institute for Chemical Research, Kyoto University Uji Kyoto 611-0011 Japan yasujiro@scl.kyoto-u.ac.jp

## Abstract

Two open-[60]fullerene–aniline conjugates were synthesized, in which the two-fold addition of diamine gave a thiazolidine-2-thione ring on the [60]fullerene cage in the presence of CS_2_. By increasing the number of *N*,*N*-dimethylaniline moieties, the absorption edge was considerably shifted up to 1200 nm owing to effective acceptor–donor interactions.

## Abstract 2

Two open-[60]fullerene–aniline conjugates were synthesized, where the π-conjugation is connected by an imino or amino group, and found to exhibit absorption bands tailing to 1100 and 1200 nm, respectively.

## Graphical abstract

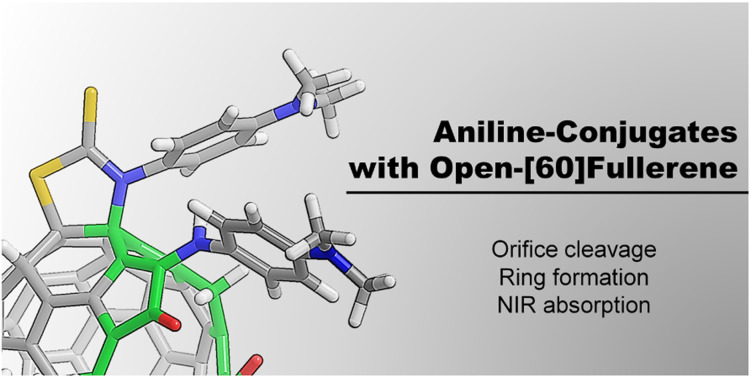

π-Extended [60]fullerenes^1^ synthesized by unidirectional π-elongation from an orifice edge could be regarded as fullerene–nanographene hybrids which have received growing attention as novel inter-carbon allotropes owing to their characteristic properties originating from both spherical and planar π-conjugated systems.^2^ Such π-extended fullerenes are readily synthesized by a reaction with aromatic 1,2-diamines which are then incorporated into the [60]fullerene π-skeleton as fused pyrazines or imidazoles whilst losing their original donor character.^1,3^ Hence, π-extended [60]fullerenes could not attain strong donor–acceptor interactions^4^ within the molecules (Fig. 1).

**Fig. 1:**
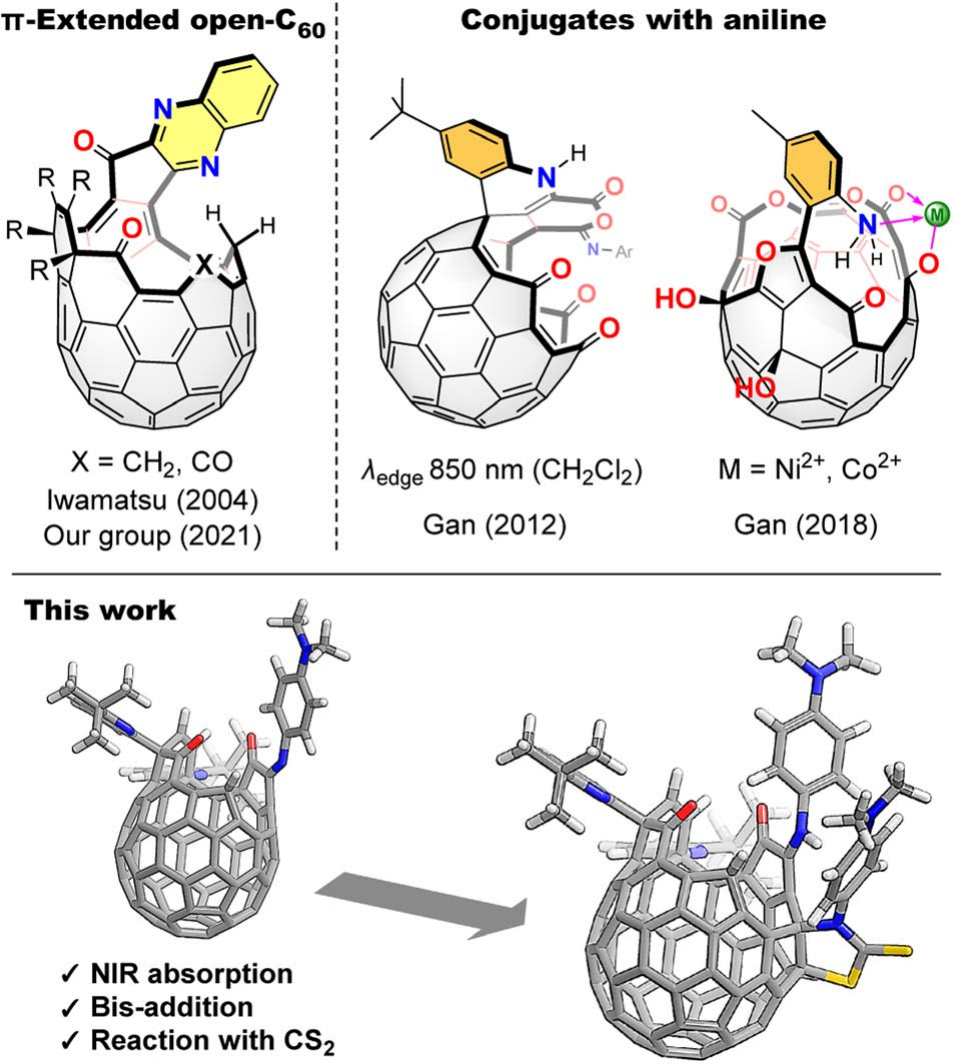
π-Extended open-[60]fullerenes and open-[60]fullerene–aniline conjugates.

Contrastingly, open-cage [60]fullerene derivatives^5^ with a conjugated aniline are regarded as [60]fullerene-based near-infrared (NIR) dyes, reflecting the strong donor nature of the aniline moiety. For example, an open-cage [60]fullerene derivative possessing a 1,4-dihydroquinoline moiety introduced as a donor unit at the orifice edge exhibits an NIR absorption band which tails to 850 nm in CH_2_Cl_2_ as found by Gan and co-workers in 2012 (Fig. 1).^6^ Recently, we reported a dehydrative condensation of a carbonyl group on the [60]fullerene orifice with *o*- and *p*-substituted aromatic diamines, which afford NIR-absorbing dyes showing absorption edges at 900 nm (benzonitrile)^7^ and 1050 nm (toluene),^8^ respectively. In these molecules, the aniline moiety is conjugated with the [60]fullerene skeleton by an imino group. Herein, we call these materials as open-[60]fullerene–aniline conjugates, in which their conjugation is connected by a single bond or certain linker between the two units. Notably, such open-[60]fullerene–aniline conjugates can act as ligands^9^ toward metals, *i.e.*, Ni^2+^ and Co^2+^, as reported by Gan and co-workers in 2018.^10^ However, open-[60]fullerene–aniline conjugates have been, to the best of our knowledge, limited to only five examples as shown above. Therefore, a synthetic variety of the conjugates is under development with leaving its understanding still elusive. In this paper, we report a reaction with *N*,*N*-dimethyl-1,4-phenylenediamine, yielding two open-[60]fullerene–aniline conjugates, both of which exhibit NIR absorption reaching to 1100 and 1200 nm, respectively (Fig. 1).

The reaction of 1^11^ with 10 equiv. of *N*,*N*-dimethyl-1,4-phenylenediamine was conducted in *o*-dichlorobenzene (ODCB) at room temperature for 2 h (Fig. 2a). As the result, open-[60]fullerene–aniline conjugate 2 was obtained in 50% isolated yield. In this reaction, there are two double bonds to be possibly cleaved, *i.e.*, C(1)=C(2) and C(3)=C(4). According to our previous study on a reaction of 1 with *o*-phenylenediamine,^7^ a cleavage of the C(3)=C(4) bond is unable to proceed due to a steric demand. In addition, NMR spectral feature of 2 showed a close resemblance with that for the product obtained by the reaction with *o*-phenylenediamine. Therefore, 2 was characterized to have a 16-membered-ring orifice in which the reaction took place on the C(1)=C(2) bond. We also obtained another aniline-conjugate 3 in 15% yield. The molecular ion peak of 3 was detected at *m*/*z* 1418.3493 which is identical to [2 + diamine + CS_2_]˙^−^. The origin of CS_2_ is the solvent used for loading the sample to a silica gel column in the purification step. The control experiment confirmed that the conversion of 2 into 3 proceeds upon exposure to CS_2_ in the presence of *N*,*N*-dimethyl-1,4-phenylenediamine at room temperature. Bis-adduct 3 was, however, relatively labile at room temperature so that it was slowly decomposed into 2 by a loss of the second aniline and CS_2_ even in a solid state. To examine the thermal stability of 3, multiple reaction monitoring was conducted for the isolated signal at *m*/*z* 1419.3486 ([3 + H]^+^) by MS/MS. Below collision energy of 35 eV, only one signal at *m*/*z* 1419.3486 was observed. By increasing the energy level up to 70 eV, however, the isolated chemical species was partly decomposed with showing a fragment peak at *m*/*z* 1207.3068 which is assignable to [2 + H]^+^ though no signals were observed at 100 eV. These results indicate that 3 could be thermally transformed into 2.

**Fig. 2:**
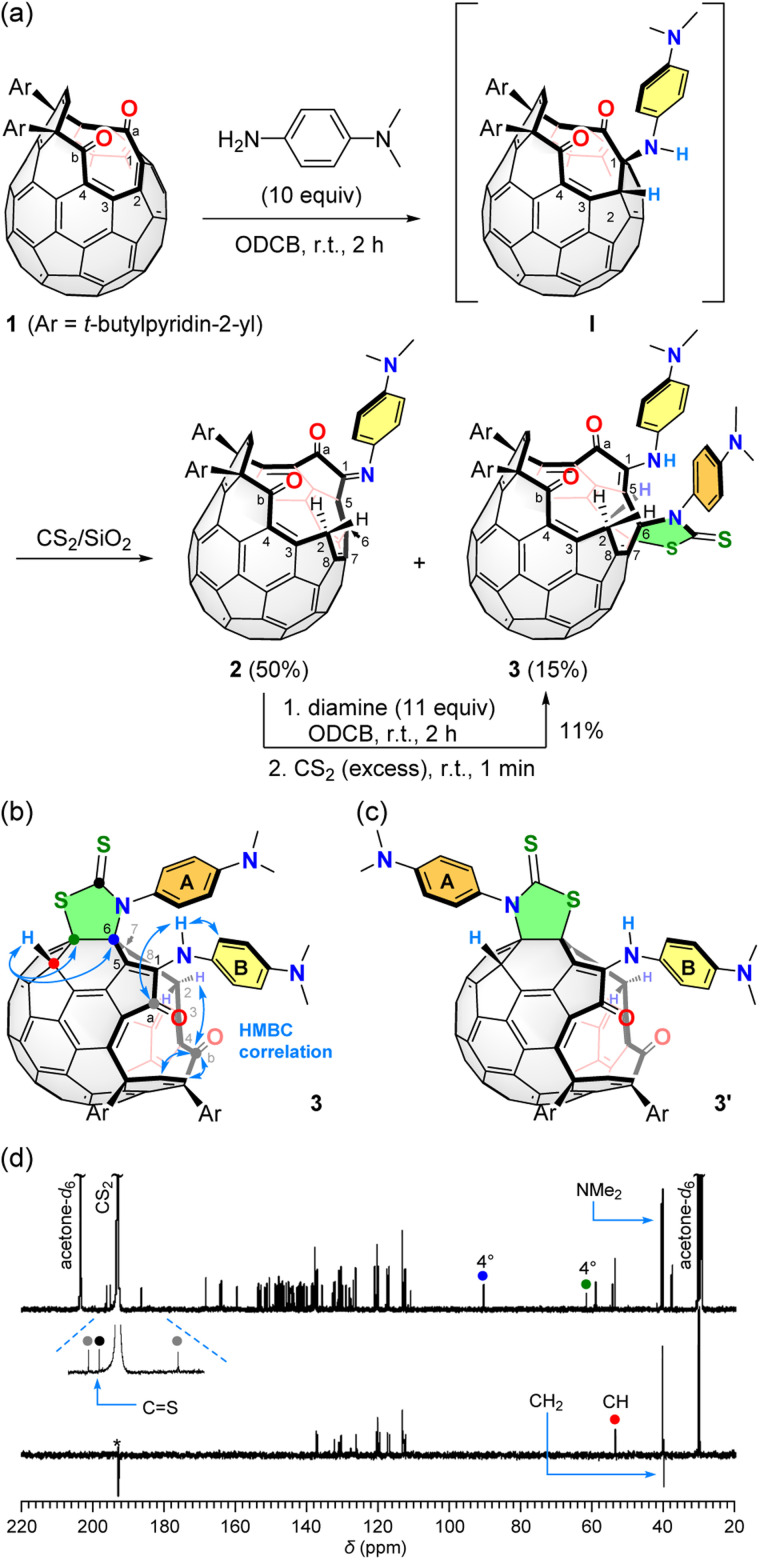
(a) Synthesis of 2 and 3. (b) HMBC correlation of 3. (c) Structure of possible isomer **3′**. (d) ^13^C NMR and DEPT 135 spectra (201 MHz, acetone-*d*_6_/CS_2_ (1 : 5)) of 3. The asterisk denotes artefact.

The long-range correlation of C(b)⋯H(2) and C(a)⋯NH observed for 3 by HMBC (Fig. 2b) further supports the cleavage of the C(1)=C(2) bond both for 2 and 3 since these two compounds are interconvertible. For the full characterization of 3, we subsequently performed DEPT and HSQC measurements confirming the presence of a methine group (*δ* 53.94 ppm) which has a long-range correlation with two quaternary carbons found at *δ* 90.69 and 61.94 ppm as judged from the HMBC measurement (Fig. 2b). The ^13^C NMR spectrum (201 MHz, acetone-*d*_6_/CS_2_ (1 : 5)) of 3 showed three signals at *δ* 196.35, 195.17, and 186.60 ppm, which were assigned to be two carbonyl and one thiocarbonyl groups (Fig. 2d). From these results, the structure of 3 was characterized to possess a thiazolidine-2-thione ring^12*a*^ though the ring position could not be determined experimentally. So, we performed theoretical calculations for the most probable structures 3 and **3′** (Fig. 2c). As the results, 3 was suggested to be more stable by Δ*E* −1.4 kcal mol^−1^ than **3′** (B3LYP-D3/6-31G(d)).

Upon seeing ^1^H NMR spectrum (500 MHz, acetone-*d*_6_/CS_2_ (1 : 5)) of 3, the proton signals corresponding to ring A was found to be anti-symmetrized at room temperature while only two doublet signals were observed for ring B (Fig. 3a). This is indicative of the restricted ring-rotation of A. By increasing the temperature up to 40 °C, the proton signals of ring A became broadened owing to the ring-rotation occurring on a time scale closer to NMR acquisition time. Such restriction of the phenyl ring could be considered only for the structure of 3 in which two phenyl rings are closely located while the two phenyl rings in **3′** are separately arranged. This strongly supports the regiochemistry of the thiazolidine-2-thione ring, excluding alternative structural isomers.

**Fig. 3:**
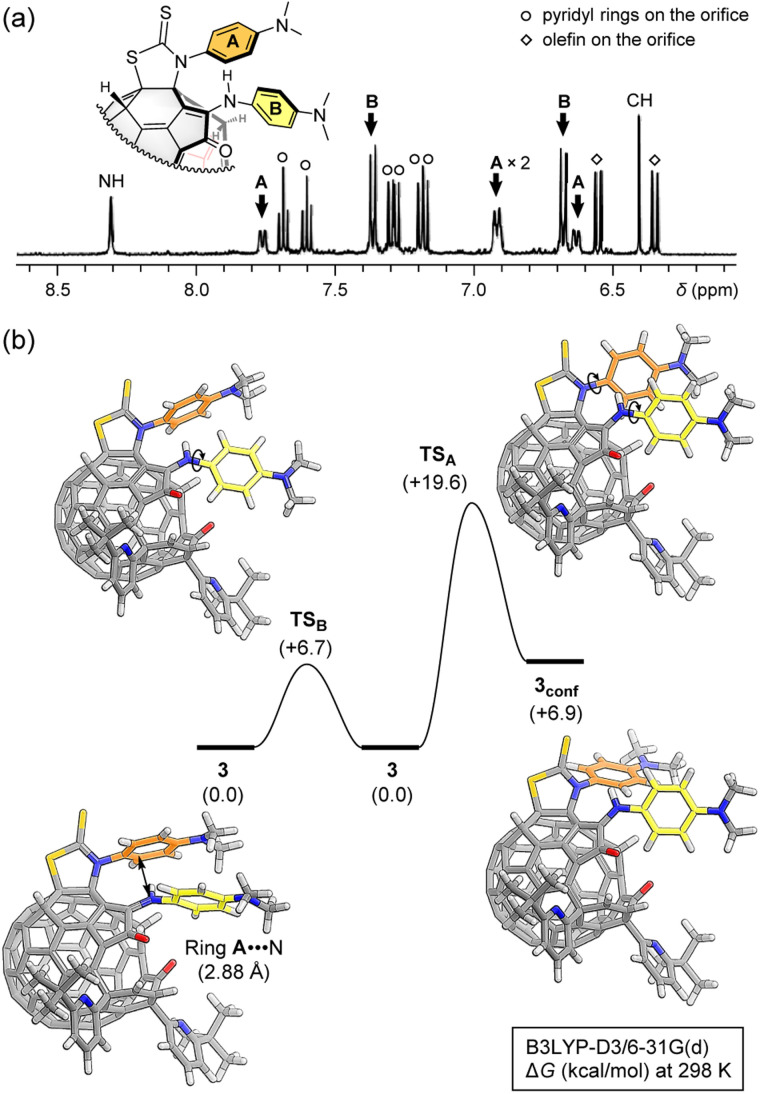
(a) ^1^H NMR spectrum (500 MHz, acetone-*d*_6_/CS_2_ (1 : 5), 20.4 °C) of 3. (b) Rotation barriers of rings A and B (B3LYP-D3/6-31G(d)).

According to theoretical calculations (B3LYP-D3/6-31G(d)), ring A faces to an N-atom of the enamine moiety in close proximity with a contact distance of 2.88 Å which is rather shorter than the sum of van der Waals radii of C- and N-atoms (3.25 Å)^13^ (Fig. 3b). This might contribute to the restricted rotation of ring A. Then, we computed barriers of the ring-rotation (B3LYP-D3/6-31G(d)). The rotation of ring A induces a ring-rotation of B, leading to a conformational change of ring B, which gives 3_conf_*via* an activation barrier of Δ*G*^‡^ +19.6 kcal mol^−1^ (Fig. 3b). This is considerably higher than that of ring B alone (+6.7 kcal mol^−1^). These ring-rotation dynamics are reminiscent of molecular machines.^14^ Since 3_conf_ stands at a higher energy level of Δ*G* +6.9 kcal mol^−1^, only conformation 3 might be observed under the thermal equilibrium at room temperature, thus showing anti-symmetrized signal pattern for ring A.

It is well-known that the reaction of primary amine with CS_2_ affords corresponding dithiocarbamic acid.^12^ In our reaction system, however, the addition of the dithiocarbamic acid to 2 unlikely occurs since it requires a Cu(ii)/O_2_ catalyst for the more simple case of pristine [60]fullerene.^12*a*^ Therefore, 3 is considered to be formed *via* an alternative scheme (Fig. 4a). In the initial step, the first *N*,*N*-dimethyl-1,4-phenylenediamine molecule undergoes a nucleophilic addition to 1 at the conjugated olefin moiety which has large LUMO coefficients (B3LYP-D3/6-31G(d)) (Fig. 4b). The thus-formed intermediate (I) is then transformed into 2 by a C–C bond cleavage. The second *N*,*N*-dimethyl-1,4-phenylenediamine molecule nucleophilically attacks to 2 at the α,β-unsaturated imine moiety which has again large LUMO coefficients (Fig. 4b), giving bis-adduct II.

**Fig. 4:**
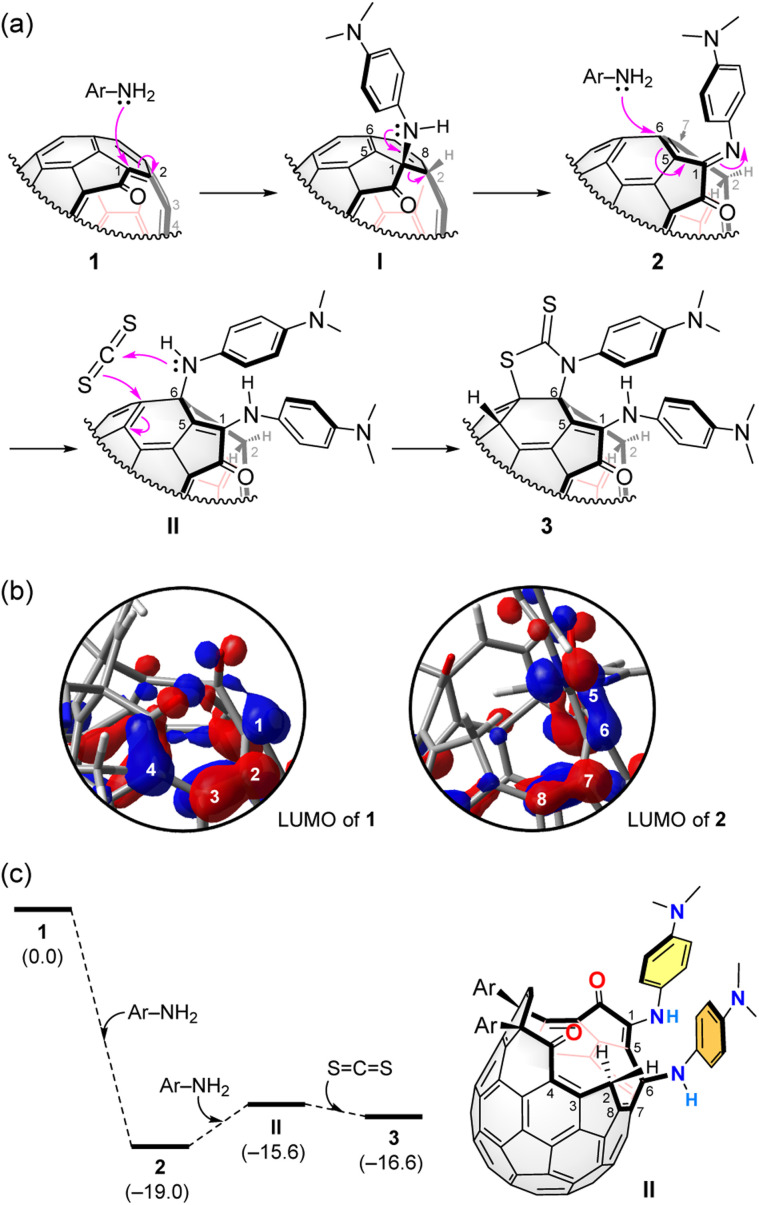
(a) Plausible mechanism. (b) The LUMOs of 1 and 2 (B3LYP-D3/6-31G(d)). (c) Energy profile (B3LYP-D3/6-31G(d)). The Δ*G* values at 298 K were shown in parentheses.

Subsequently, the reaction with CS_2_ furnishes 3 possessing a thiazolidine-2-thione ring. The similar reaction of secondary amines and CS_2_ was reported recently.^12*b*^ The energy profile of the conversion of 1 into 3 was computed (Fig. 4c). Open-[60]fullerene–aniline conjugate 2 is suggested to be more stable by Δ*G* −19.0 kcal mol^−1^ than 1 at 298 K (B3LYP-D3/6-31G(d)). The reaction with the second diamine molecule, however, destabilizes the product (II) by Δ*G* +3.4 kcal mol^−1^ while the further reaction with CS_2_ gives 3 which attains a stabilization energy of Δ*G* −1.0 kcal mol^−1^ relative to II. So far, we have not succeeded in isolating II. From the fact that the conversion of 3 into 2 gradually proceeded even at ambient conditions, II which is less stable than 3 might be hardly isolated.

To examine the effect of conjugation with the aniline moiety on electronic properties of **1–3**, we measured UV-vis-NIR absorption spectra and cyclic voltammograms (Fig. 5). As shown in Fig. 5a, 2 and 3 have large absorption coefficients over a wide range covering visible to NIR region. Notably, they exhibit strong NIR absorption bands which are absent in 1. Accordingly, the solution colour varies from yellow (1) to green (2) and brown (3). The absorption edges are bathochromically shifted in the order of 1 (870 nm) < 2 (1100 nm) < 3 (1200 nm), reflecting a count of conjugated aniline moieties. This absorption edge extends beyond the NIR-I region (700–950 nm) into the NIR-II region (1000–1700 nm). Since solvatochromism was observed for 2 over the measured range (Fig. S9†), the transitions have a charge-transfer character. Considering the characteristic absorption behaviour, open-[60]fullerene–aniline conjugates could be used for organic solar cells^15*a*^ and perovskite solar cells.^15*b*^ Upon seeing cyclic voltammograms (Fig. 5b), the oxidation potentials *E*_ox,pa_ of 2 (+0.44 V) and 3 (+0.26 V) were cathodically shifted by Δ*E* −0.92 and −1.10 V with respect to 1 (+1.36 V). This is in sharp contrast to the reduction potentials without showing considerable shifts. These observations suggest that the conjugation with the aniline moiety renders the HOMO levels higher while it has less contribution to the LUMO levels. The oxidation wave found at *E*_ox,pa_ −0.47 V might be attributed to S-containing compounds^16^ partly decomposed from 3*via* electrochemical process. According to theoretical calculations (B3LYP-D3/6-31G(d)), the LUMO delocalizes over the entire [60]fullerene skeleton for all cases whereas high HOMO coefficients were found at the *N*,*N*-dimethylaniline moieties for 2 and 3 (Fig. 5c). Thus, the electrochemical oxidation of 2 and 3 is considered to generate the corresponding radical cations at the aniline moiety while the reduction produces radical anions at the [60]fullerene cage.

**Fig. 5:**
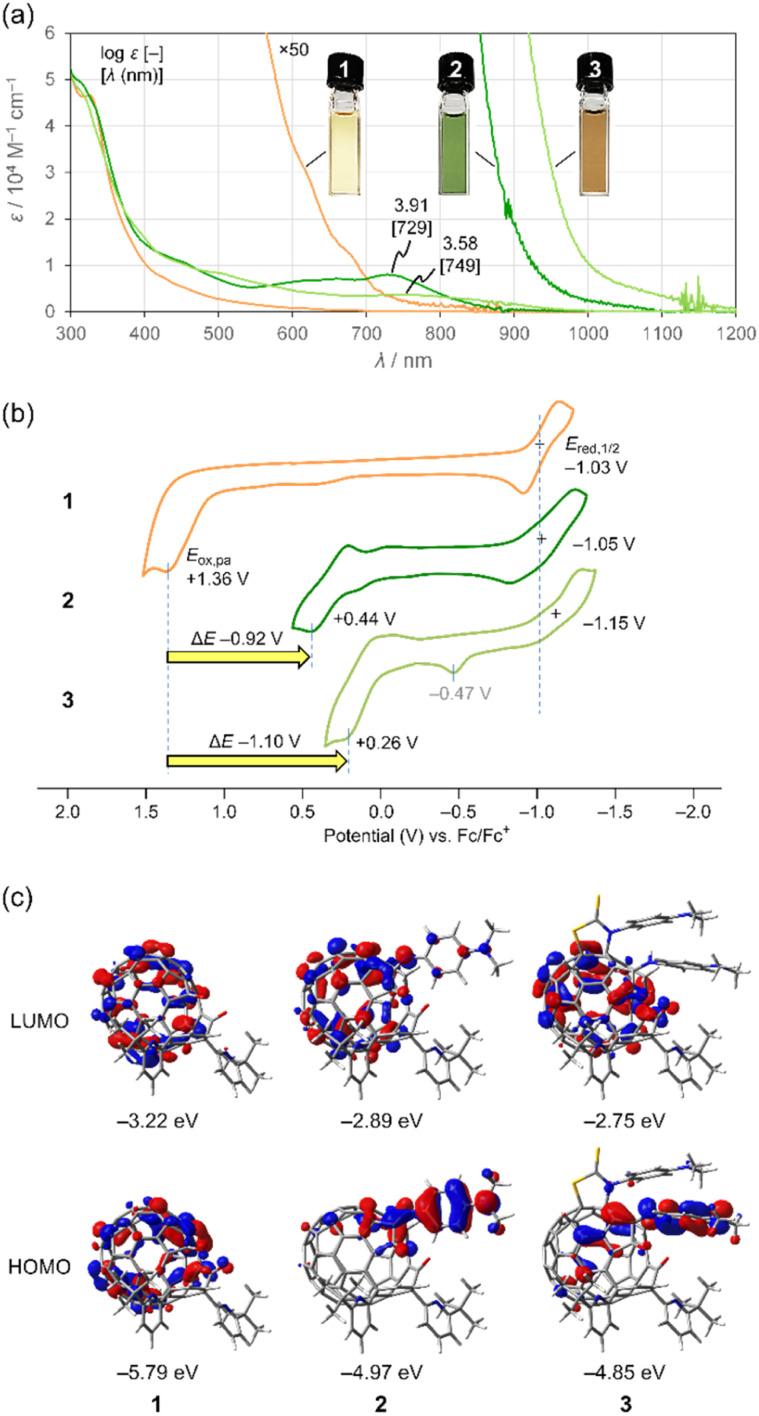
(a) UV-vis-NIR absorption spectra of **1–3** (50 μM in benzene). (b) Cyclic voltammograms of **1–3** (1 mM in ODCB, 0.1 M *n*-Bu_4_N·BF_4_, 100 mV s^−1^). (c) Molecular orbitals of **1–3** (B3LYP-D3/6-31G(d)).

In summary, we synthesized two open-[60]fullerene–aniline conjugates, 2 and 3, by the reaction of 1 with *N*,*N*-dimethyl-1,4-phenylenediamine. Bis-adduct 3 contains a fused thiazolidine-2-thione ring which was generated from 2 in the presence of the diamine and CS_2_ under catalyst-free conditions. The rotation of one of the two *N*,*N*-dimethylaniline moieties in 3 was found to be severely restricted due to a steric demand as confirmed by NMR spectroscopy. The introduction of *N*,*N*-dimethylaniline caused a significant cathodic shift of oxidation potentials for 2 (*E*_ox,pa_ +0.44 V) and 3 (+0.26 V), as well as a bathochromic shift of absorption bands tailing to 1100 and 1200 nm, respectively. These findings demonstrated herein would facilitate the access to [60]fullerene-based NIR dyes with a wide structural variety when replacing aniline with other donors.

## Conflicts of interest

There are no conflicts to declare.

## Supplementary Material

RA-013-D3RA02113K-s001
