# Supplementary material for: Open-[60]fullerene–aniline conjugates with near-infrared absorption
Source: RSC Adv. 2023 May 12;13(21):14575–9. doi: 10.1039/d3ra02113k (PMC10177950; doi:10.1039/d3ra02113k)
Supplement: RA-013-D3RA02113K-s001 [file RA-013-D3RA02113K-s001.pdf]

Electronic Supplementary Material (ESI) for RSC Advances.
This journal is © The Royal Society of Chemistry 2023

Supporting Information

Open-[60]fullerene–aniline conjugates with near-infrared absorption

Shumpei Sadai, Yoshifumi Hashikawa, and Yasujiro Murata*

Institute for Chemical Research, Kyoto University, Uji, Kyoto 611-0011, Japan
Fax: (+81)774-38-3178
E-mail: yasujiro@scl.kyoto-u.ac.jp

Contents

1. General S3
2. Computational Methods S3
3. Synthesis of **2** and **3** S4
4. UV-Vis-NIR Absorption Spectra S14
5. DFT Calculations S15
   5.1. Energy Profiles and Molecular Orbitals S15
   5.2. Rotation of Phenylene Rings in **3** S23
6. IR Spectra S29
7. Structural Isomer **3’** S30
8. References S32

1. General

The ^{1}H and ^{13}C NMR measurements were carried out with JEOL JNM ECA500 and Bruker Avance III 800US Plus instruments at room temperature unless otherwise noted. The NMR chemical shifts were reported in ppm with reference to residual protons and carbons of $CDCl_3$ (δ 7.26 ppm in ^{1}H NMR, δ 77.00 ppm in ^{13}C NMR) and acetone-d_6 (δ 2.05 ppm in ^{1}H NMR, δ 29.92 ppm in ^{13}C NMR). APCI (atmospheric pressure chemical ionization) mass spectra were measured on a Bruker micrOTOF-Q II. IR spectra were taken with a Shimadzu IR-Affinity 1S. UV-vis-NIR absorption spectra were measured with a Shimadzu UV-3150 spectrometer. Cyclic voltammetry was conducted on a BAS Electrochemical Analyzer ALS620C using a three-electrode cell with a glassy carbon working electrode, a platinum wire counter electrode, and an $Ag/AgNO_3$ reference electrode. The measurements were carried out under N_2 atmosphere using *o*-dichlorobenzene (ODCB) solutions of 1.0 mM samples and 0.10 M tetrabutylammonium tetrafluoroborate (n-$Bu_4N \cdot BF_4$) as a supporting electrolyte. The redox potentials were calibrated with ferrocene used as an internal standard which was added after each measurement. The high-performance liquid chromatography (HPLC) was performed with the use of a Cosmosil Buckyprep column (250 mm in length, 4.6 mm in inner diameter) for analytical purpose. Thin layer chromatography (TLC) was performed on glass plates coated with 0.25 mm thick silica gel 60F-254 (Merck). Column chromatography was performed using PSQ 60B (Fuji Silysia).

Fullerene C_{60} was purchased from SES Research Co. Carbon disulfide and ethyl acetate were purchased from FUJIFILM Wako Pure Chemical Corporation. *N*,*N*-Dimethyl-1,4-phenylenediamine was purchased from Tokyo Chemical Industry Co. Ltd. Toluene was purchased from Nacalai Tesque, Inc. ODCB was purchased from Sigma-Aldroch Co. LLC.

All reactions were carried out under Ar atmosphere. Unless otherwise noted, materials purchased from commercial suppliers were used without further purification. Compound **1**[1] was synthesized according to literature procedures.

2. Computational Methods

All calculations were conducted using the Gaussian 09 program. All structures at the stationary and transition states were optimized at the B3LYP-D3/6-31G(d) level of theory and confirmed by the frequency analyses at the same level of theory.

3. Synthesis of 2 and 3

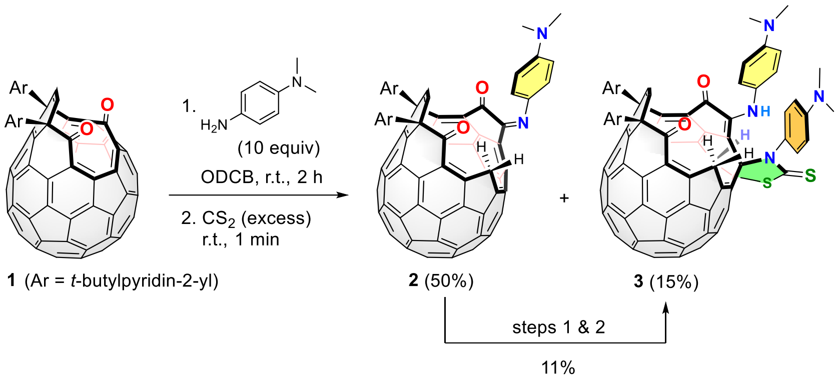

[Reaction with **1** and the diamine]

Powdery **1** (20.0 mg, 18.7 μmol) and *N*,*N*-dimethyl-1,4-phenylenediamine (25.5 mg, 187 μmol, 10.0 equiv) were placed into a Schlenk tube and degassed through three vacuum–Ar cycles. ODCB (1.00 mL) was added and the resulting solution was stirred at room temperature for 2 h. After the reaction, CS_2 (ca. 10 mL) was added and the solution was kept at room temperature for 1 min. The crude mixture was then purified by column chromatography using silica gel (toluene and then toluene/ethyl acetate (100:1) to (50:1)) to give unreacted **1** (2.56 mg, 2.39 μmol, 12%), **3** (4.06 mg, 2.86 μmol, 15%), and **2** (11.3 mg, 9.38 μmol, 50%) as black powders.

[Conversion of **2** into **3**]

Powdery **2** (5.02 mg, 4.14 μmol) and *N*,*N*-dimethyl-1,4-phenylenediamine (6.38 mg, 46.8 μmol, 11.3 equiv) were placed into a Schlenk tube and degassed through three vacuum–Ar cycles. ODCB (0.250 mL) was added and the resulting solution was stirred at room temperature for 2 h. After the reaction, CS_2 (ca. 5 mL) was added and the resulting solution was kept at room temperature for 1 min. The crude mixture was then purified by column chromatography using silica gel (toluene/ethyl acetate (50:1) to (30:1)) to give **3** (0.62 mg, 0.44 μmol, 11%) and unreacted **2** (2.12 mg, 1.76 μmol, 42%) as black powders.

2: UV-vis-NIR (benzene) λ_{max} (log ε) 729 (3.91); IR (KBr) ν 1732, 1717, 1697, 1684 (C=O, four bands were observed probably arising from two dominant conformations of **2**) cm^{-1}; ^{1}H NMR (500 MHz, $CDCl_3$) δ 8.18 (d, 2H, J = 9.2 Hz), 7.62 (t, 1H, J = 8.0 Hz), 7.57 (t, 1H, J = 8.0 Hz), 7.32 (d, 1H, J = 8.0 Hz), 7.29 (d, 1H, J = 8.0 Hz), 7.20 (d, 1H, J = 8.0 Hz), 7.143 (d, 1H, J = 10.3 Hz), 7.141 (d, 1H, J = 8.0 Hz), 6.91 (d, 1H, J = 10.3 Hz), 6.78 (d, 2H, J = 9.2 Hz), 5.01 (d, 1H, J = 20.0 Hz), 4.72 (d, 1H, J = 20.0 Hz), 3.09 (s, 3H), 1.23 (s, 9H), 1.16 (s, 9H); ^{13}C NMR (126 MHz, $CDCl_3$) δ 196.80, 186.35, 168.32, 167.95, 164.61, 162.78, 153.46, 153.30, 152.95, 151.06, 150.72, 150.14, 149.30, 149.21, 149.19, 148.47, 148.38, 148.12, 147.90, 147.76, 147.52, 147.50, 147.44, 147.29, 146.85, 146.75, 146.45, 145.94, 145.13, 145.03, 144.95, 144.76, 144.61, 144.07, 144.05, 143.98, 143.28, 142.95, 142.56, 141.50, 141.38, 140.47, 139.02, 138.62, 137.43, 137.27, 137.24, 137.15, 137.11, 136.98, 136.81, 136.78, 136.57, 136.32, 135.83, 135.62, 135.00, 134.33, 133.52, 133.35, 131.56, 131.22, 131.04, 130.13, 127.87, 127.80, 126.27, 120.17, 119.76, 117.19, 116.61, 111.94, 59.52, 55.00, 41.75, 40.26, 37.67, 37.57, 29.93, 29.83 (The sum of carbon signals must be 83 in theory. Observed 80. The 3 sp^{2} carbon signals are overlapped.); HRMS (APCI) m/z: $[M]^{\bullet-}$ Calcd for $C_{90}H_{38}N_4O_2$ (**2**) 1206.3000; Found 1206.3022.

3: UV-vis-NIR (benzene) λ_{max} (log ε) 749 (3.58); IR (KBr) ν 3323 (NH), 1717 (C=O), 1684 (C=O) cm^{-1}; ^{1}H NMR (500 MHz, acetone-d_6/CS_2 (1:5), 20.4 °C) δ 8.31 (br s, 1H), 7.76 (dd, 1H, J = 8.9, 2.6 Hz), 7.69 (t, 1H, J = 8.0 Hz), 7.60 (t, 1H, J = 8.0 Hz), 7.36 (d, 2H, J = 8.9 Hz), 7.30 (d, 1H, J = 8.0 Hz), 7.29 (d, 1H, J = 8.0 Hz), 7.19 (d, 1H, J = 8.0 Hz), 7.18 (d, 1H, J = 8.0 Hz), 6.92 (dd, 1H, J = 8.9, 2.6 Hz), 6.91 (dd, 1H, J = 8.9, 2.6 Hz), 6.68 (d, 2H, J = 8.9 Hz), 6.63 (dd, 1H, J = 8.9, 2.6 Hz), 6.55 (d, 1H, J = 10.3 Hz), 6.41 (s, 1H), 6.35 (d, 1H, J = 10.3 Hz), 4.04 (d, 1H, J = 19.5 Hz), 3.37 (d, 1H, J = 19.5 Hz), 3.09 (s, 3H), 3.00 (s, 3H), 1.36 (s, 9H), 1.26 (s, 9H); ^{13}C NMR (201 MHz, acetone-d_6/CS_2 (1:5)) δ 196.35, 195.17, 186.60, 168.42, 168.38, 164.37, 164.06, 159.84, 153.95, 153.74, 153.18, 152.97, 151.89, 151.47, 150.78, 150.72, 149.18, 148.78, 148.42, 148.15, 148.07, 148.05, 147.66, 147.50, 147.15, 147.13, 146.81, 146.78, 146.04, 145.52, 145.48, 145.36, 144.99, 144.47, 144.28, 144.16, 143.88, 143.07, 142.83, 142.65, 142.14, 142.11, 141.46, 141.14, 140.99, 140.15, 138.98, 138.79, 138.31, 138.09, 137.95, 137.86, 137.84, 137.67, 137.45, 135.89, 132.78, 132.73, 132.40, 132.23, 131.44, 131.42, 130.77, 130.51, 130.46, 129.37, 128.24, 128.07, 127.26, 126.55, 121.10, 120.57, 120.13, 117.96, 117.37, 113.65, 113.06, 112.71, 111.22, 90.69, 61.94, 59.16, 54.54, 53.94, 40.86, 40.67, 40.51,

38.10, 37.94, 30.65, 30.53 (The sum of carbon signals must be 91 in theory. Observed 91.); HRMS (APCI) *m*/*z*: $[M]^{\bullet-}$ Calcd for $C_{99}H_{50}N_6O_2S_2$ (**3**) 1418.3442; Found 1418.3493.

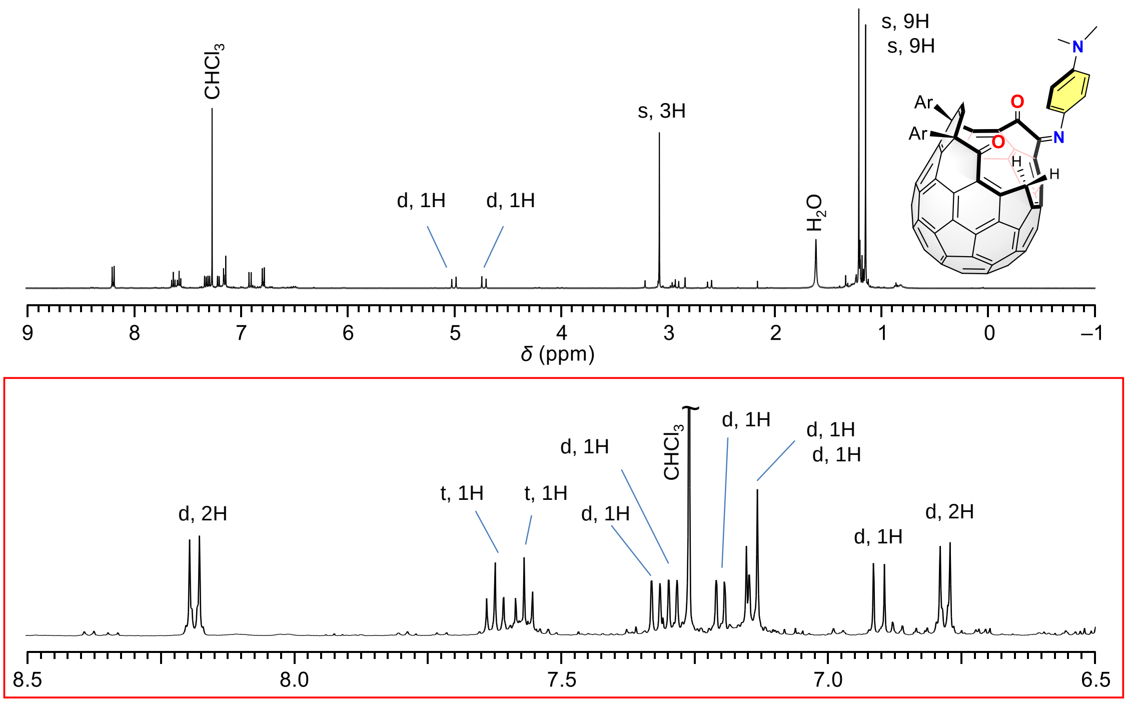

Figure S1. ^{1}H NMR spectra (500 MHz, $CDCl_3$) of **2**.

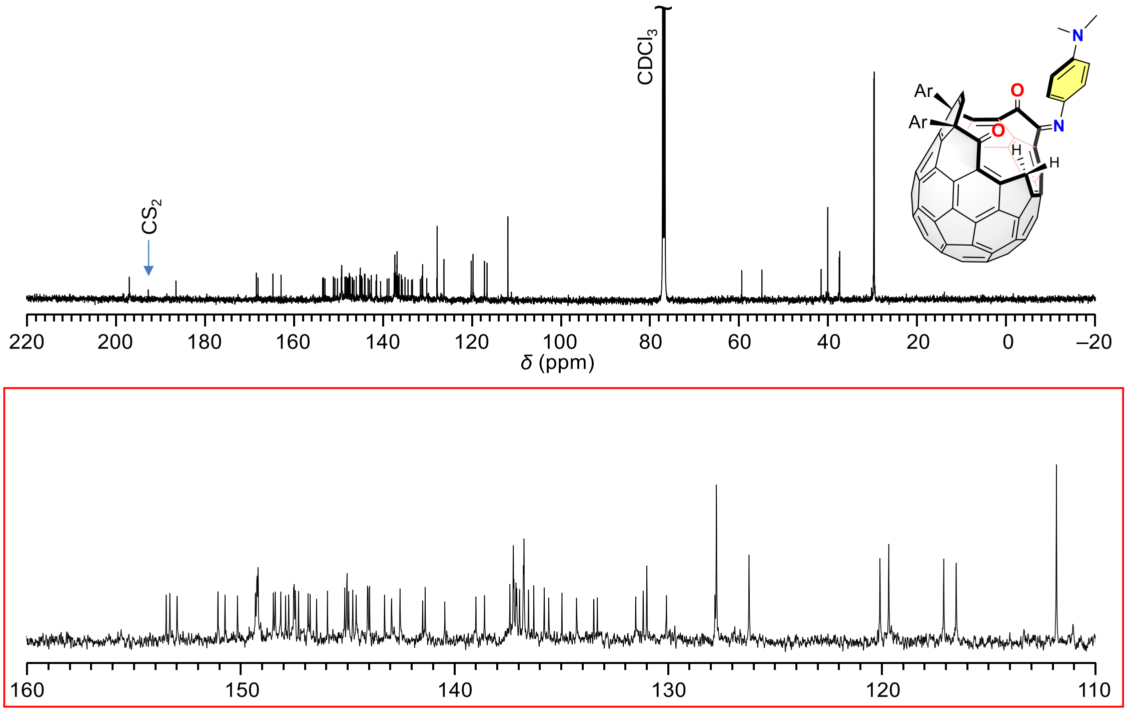

Figure S2. ^{13}C NMR spectra (126 MHz, $CDCl_3$) of **2**.

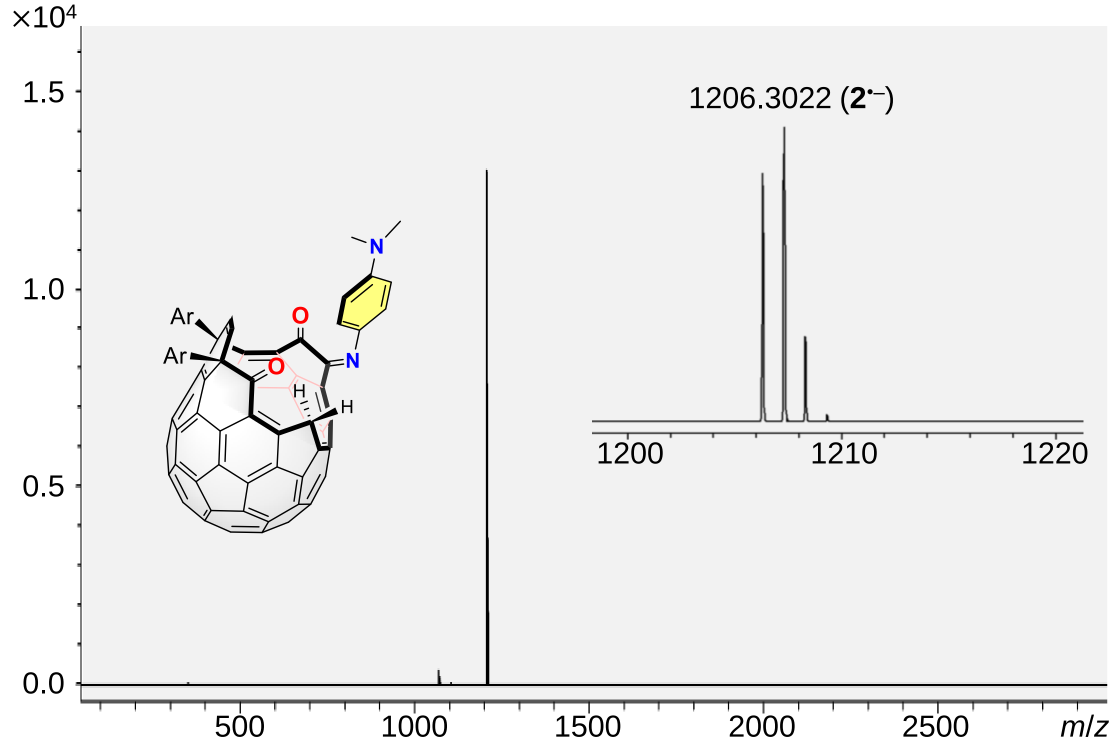

Figure S3. APCI mass spectra (negative ion mode) of **2**.

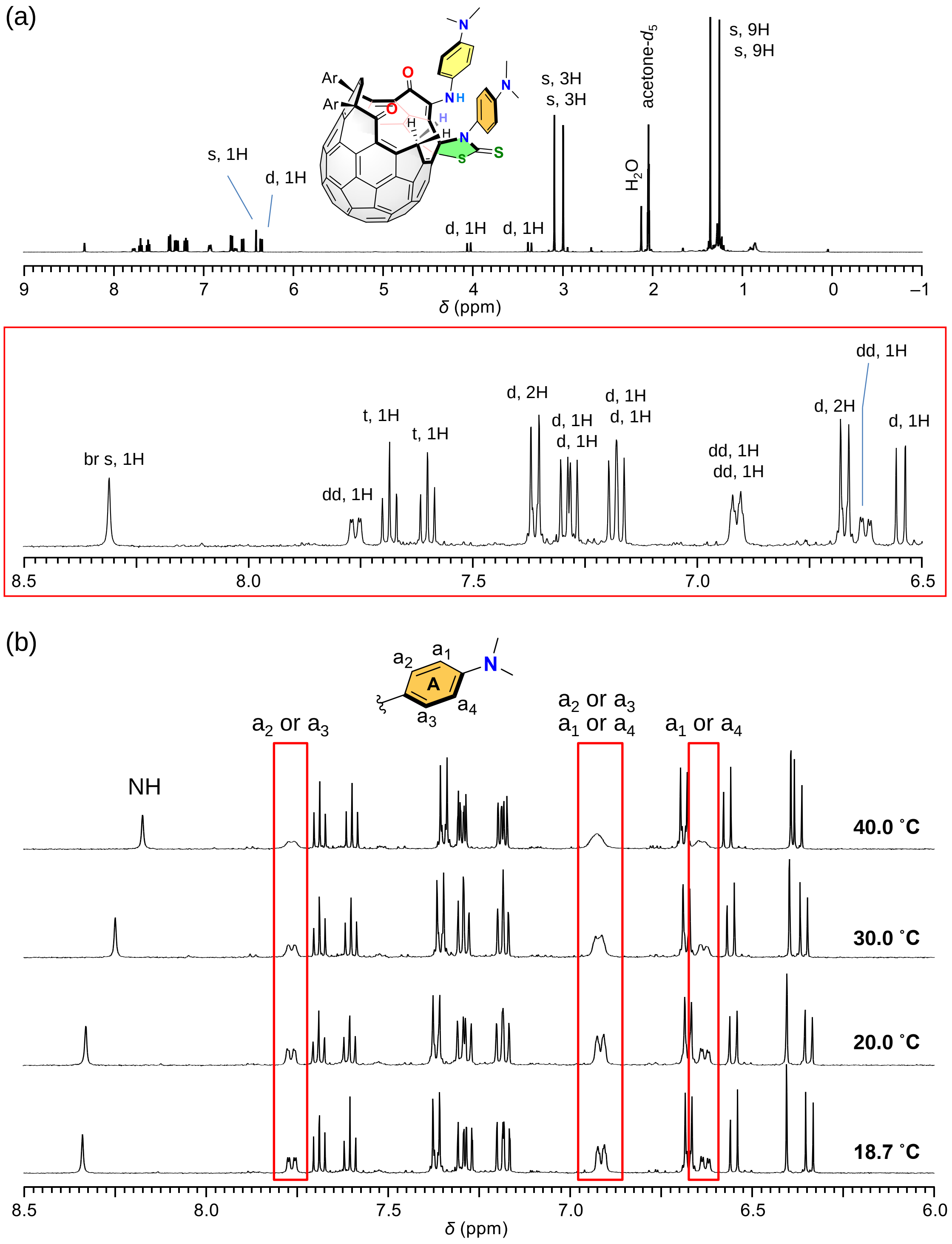

Figure S4. (a) ^{1}H NMR spectra (500 MHz, acetone-d_6/CS_2 (1:5), 20.4 °C) of **3**. (b) VT ^{1}H NMR spectra (500 MHz, acetone-d_6/CS_2 (1:5)) of the aromatic region of **3** at 18.7 °C, 20°C, 30°C, and 40°C.

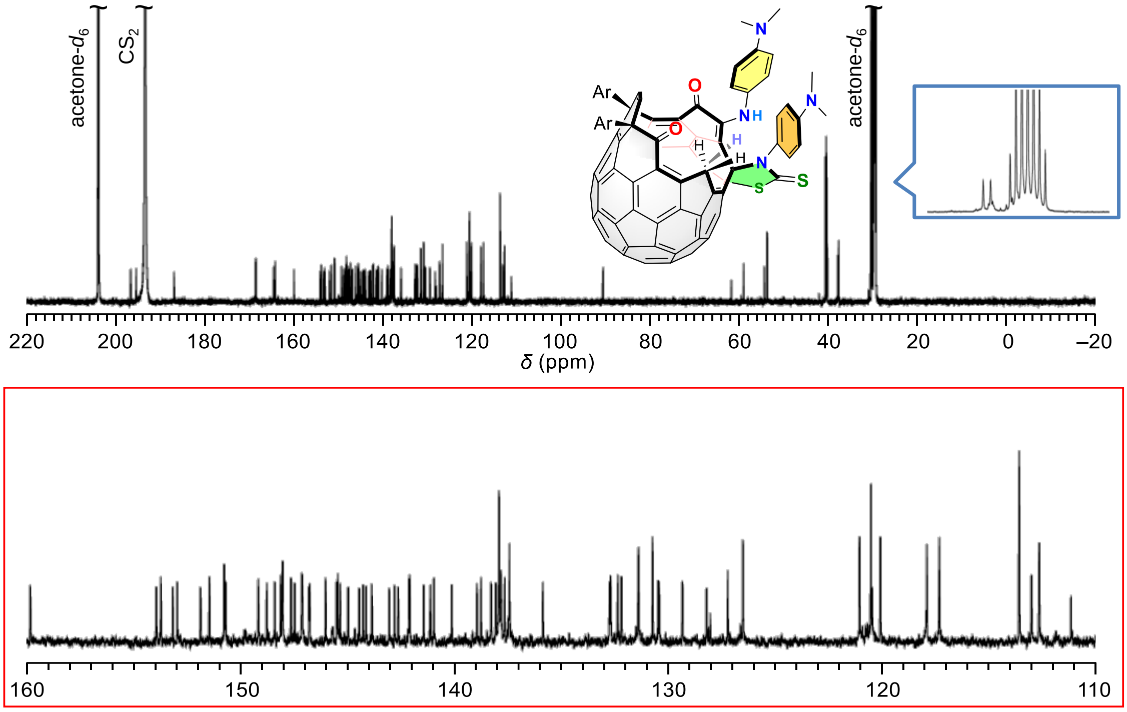

Figure S5. ^{13}C NMR spectra (201 MHz, acetone-d_6/CS_2 (1:5)) of **3**.

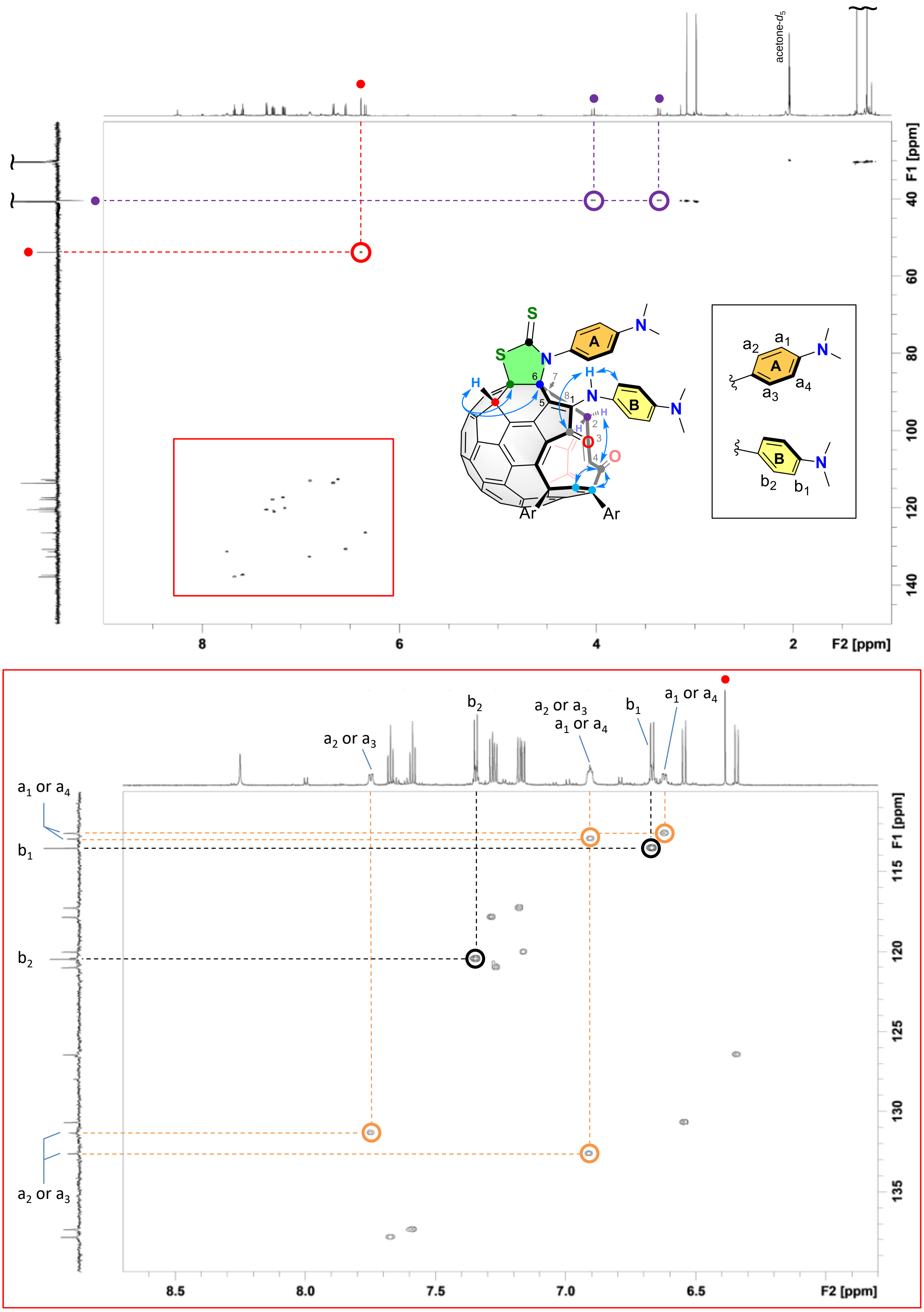

Figure S6. HSQC spectra (800 MHz, acetone-d_6/CS_2 (1:5)) of **3**.

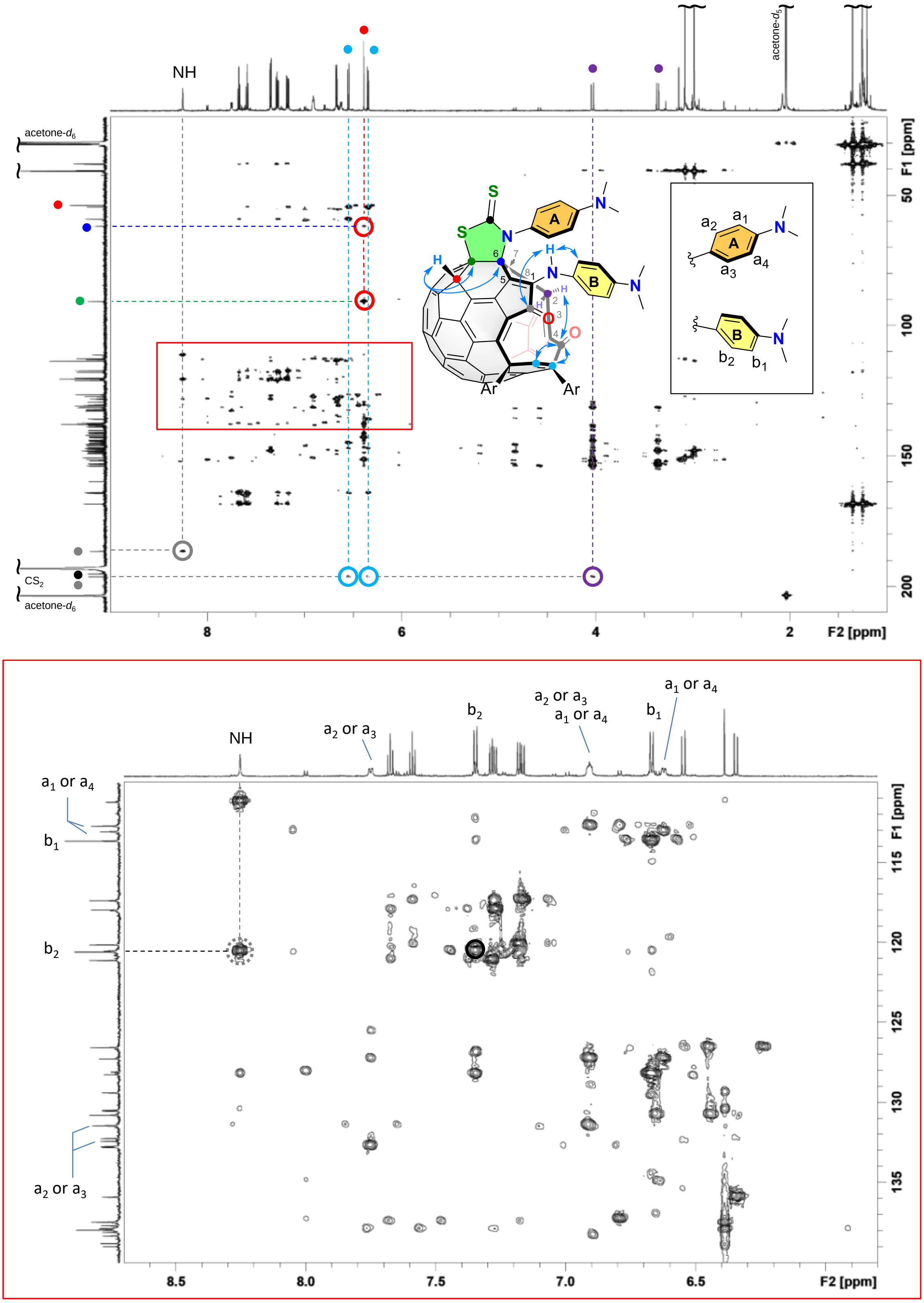

Figure S7. HMBC spectra (800 MHz, acetone-d_6/CS_2 (1:5)) of **3**.

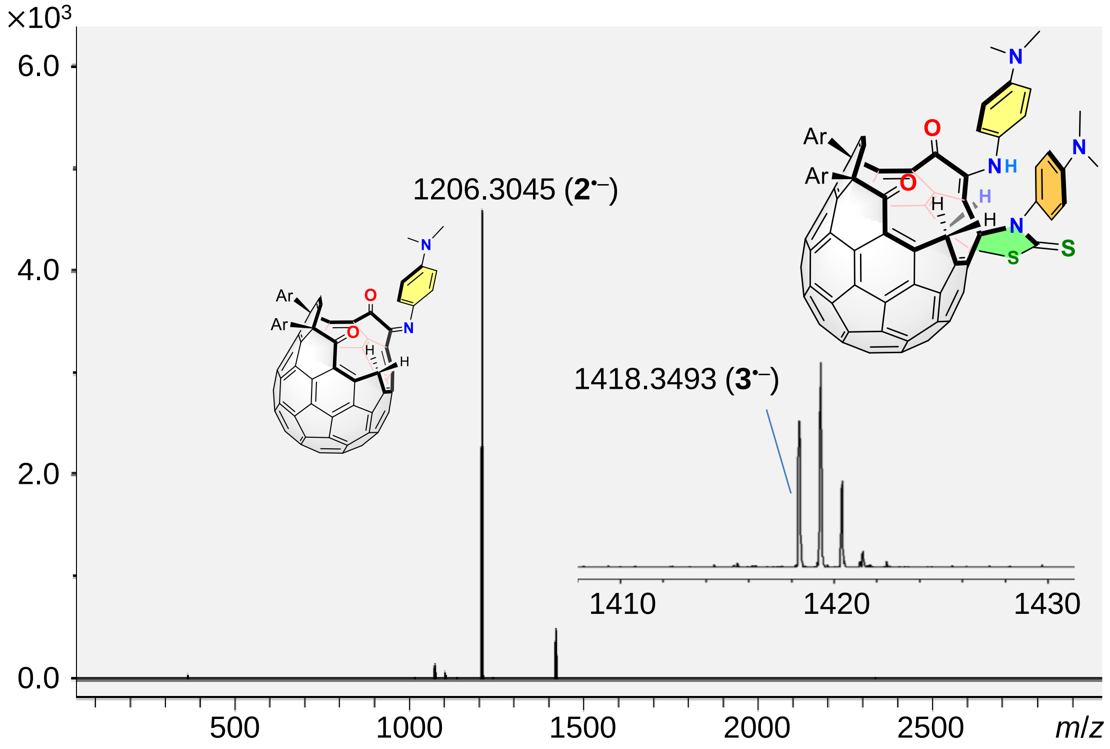

Figure S8. APCI mass spectra (negative ion mode) of **3**.

4. UV-Vis-NIR Absorption Spectra

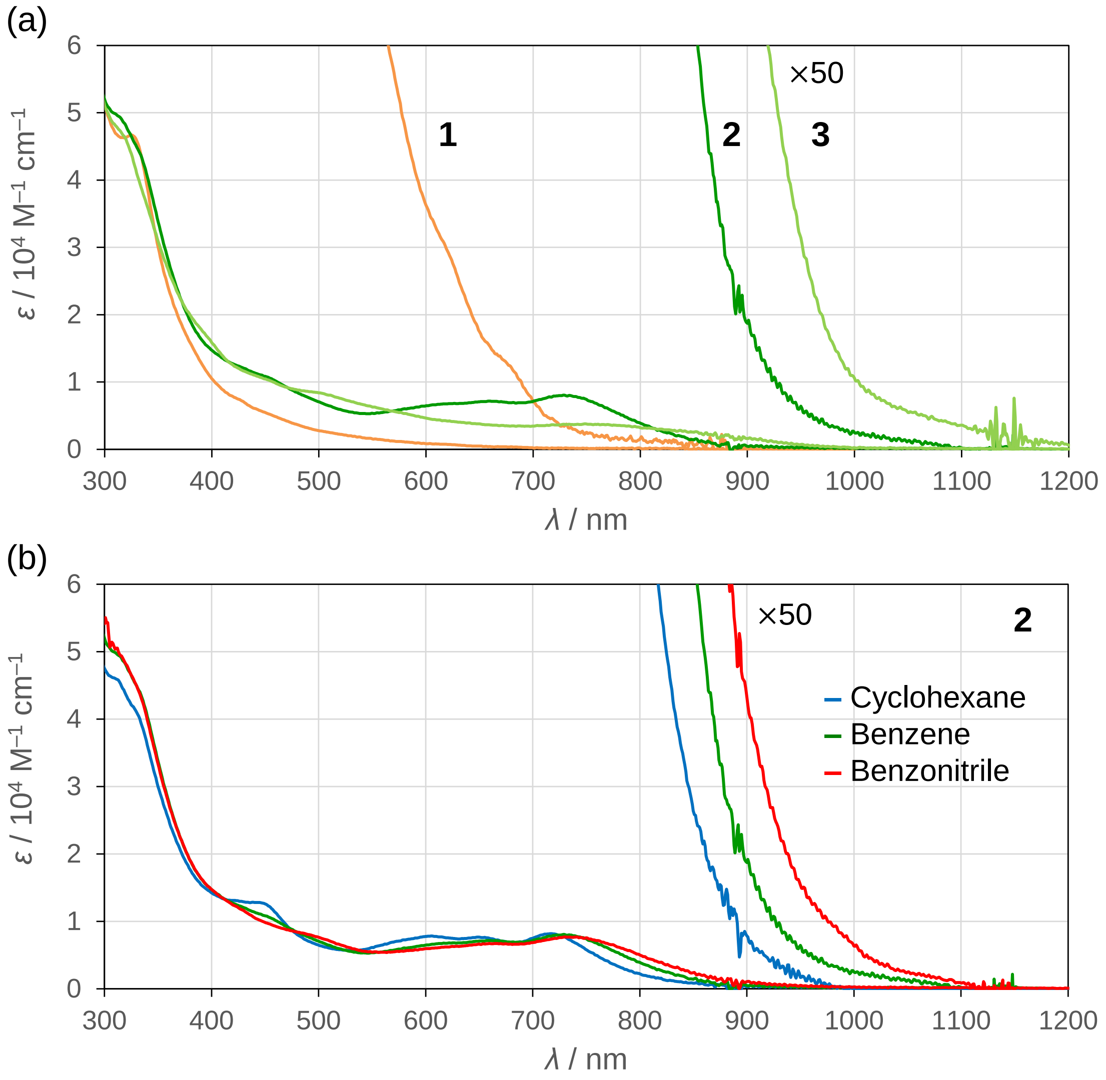

Figure S9. UV-vis-NIR absorption spectra (50 μM) of (a) **1**–**3** in benzene and (b) **2** in cyclohexane, benzene and benzonitrile.

5. DFT Calculations

5.1. Energy Profiles and Molecular Orbitals

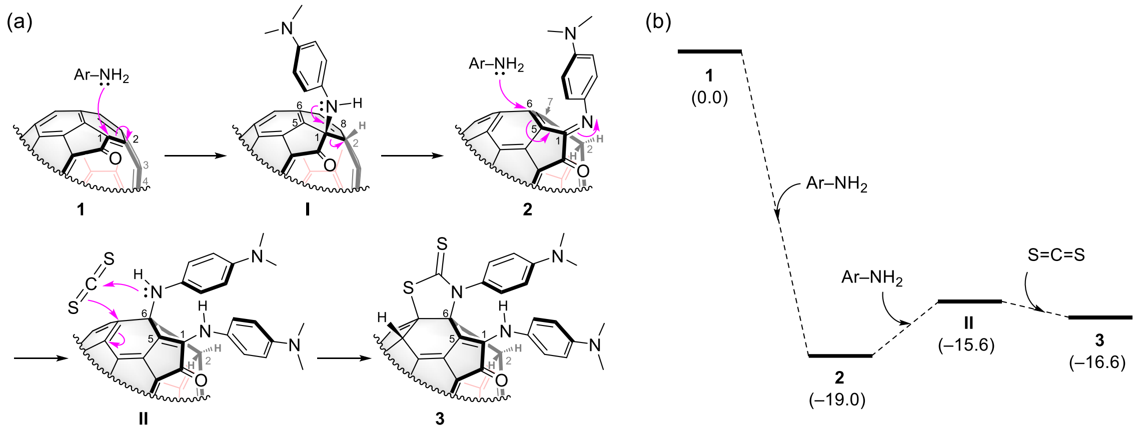

Figure S10. (a) Plausible reaction mechanism and (b) energy profile (B3LYP-D3/6-31G(d)). The Δ*G* values at 298 K were shown in parentheses.

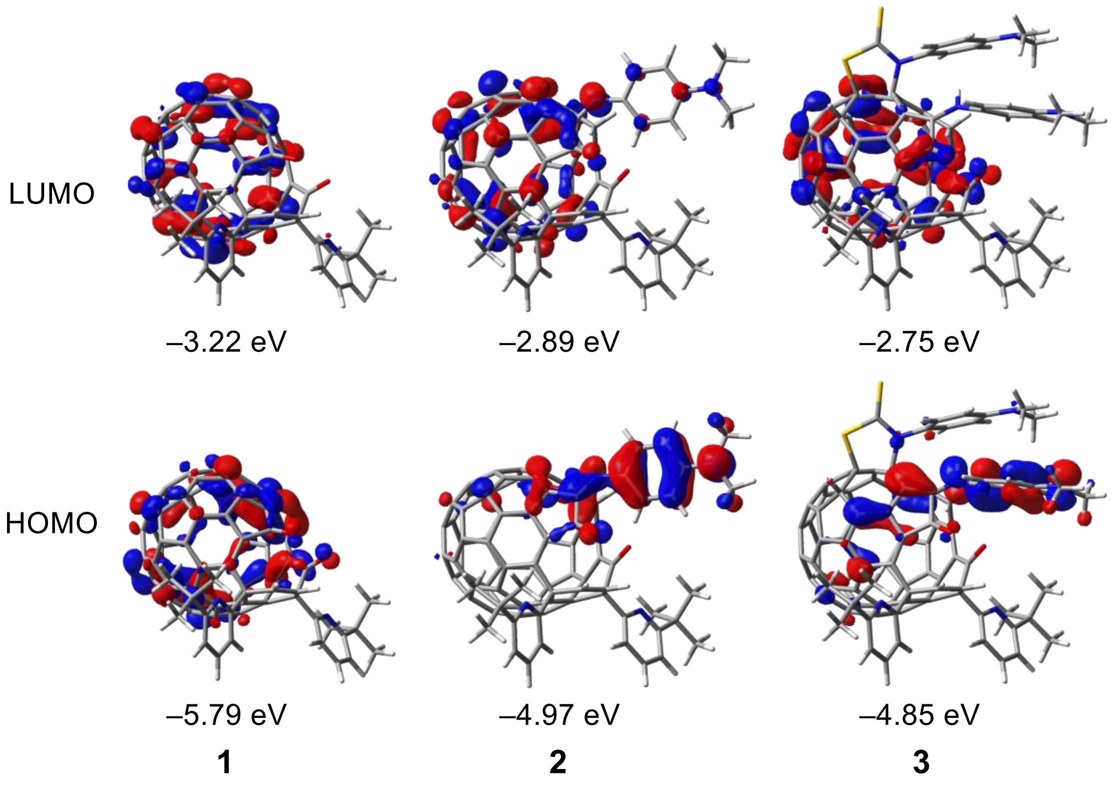

Figure S11. Molecular orbitals of **1**–**3** (B3LYP-D3/6-31G(d)).

Table S1. Optimized structure of *N*,*N*-dimethyl-1,4-phenylenediamine (B3LYP-D3/6-31G(d))

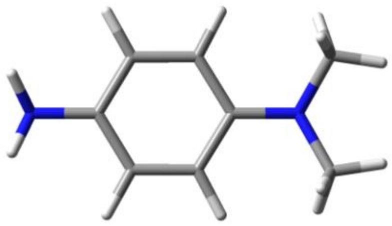

Standard orientation:

Center Number	Atomic Number	Atomic Type	Coordinates (Angstroms) X	Y	Z
1	6	0	-0.094150	-1.199648	-0.069660
2	6	0	-1.485497	-1.196260	-0.016959
3	6	0	-2.213899	-0.000000	0.008013
4	6	0	-1.485502	1.196264	-0.016998
5	6	0	-0.094156	1.199655	-0.069698
6	6	0	0.645906	0.000004	-0.110959
7	7	0	2.048638	-0.000001	-0.204060
8	7	0	-3.624015	-0.000002	-0.001890
9	6	0	2.747994	1.234266	0.109520
10	6	0	2.747969	-1.234275	0.109560
11	1	0	0.410585	-2.158357	-0.076568
12	1	0	-2.014839	-2.147017	0.006813
13	1	0	-2.014850	2.147018	0.006741
14	1	0	0.410575	2.158366	-0.076631
15	1	0	-4.029316	0.829118	0.418624
16	1	0	-4.029314	-0.829118	0.418633
17	1	0	2.473306	2.025932	-0.596713
18	1	0	2.544181	1.603113	1.130192
19	1	0	3.823967	1.071917	0.010003
20	1	0	2.544130	-1.603092	1.130238
21	1	0	2.473274	-2.025951	-0.596658
22	1	0	3.823946	-1.071944	0.010065

The total electronic energy was calculated to be –421.5802258 Hartree.

Table S2. Optimized structure of CS_2 (B3LYP-D3/6-31G(d))

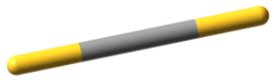

Standard orientation:

Center Number	Atomic Number	Atomic Type	Coordinates (Angstroms) X	Y	Z
1	6	0	0.000000	0.000000	0.000000
2	16	0	0.000000	0.000000	1.563660
3	16	0	0.000000	0.000000	-1.563660

The total electronic energy was calculated to be –834.4893444 Hartree.

Table S3. Optimized structure of **1** (B3LYP-D3/6-31G(d))

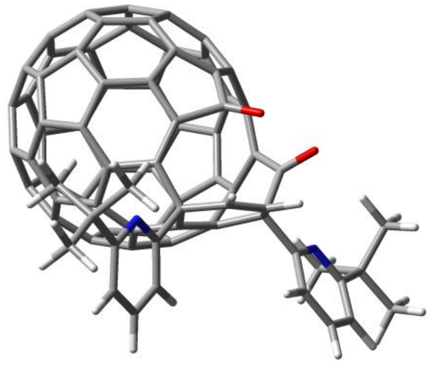

Standard orientation:

Center Number	Atomic Number	Atomic Type	Coordinates (Angstroms) X	Y	Z
1	6	0	-3.431157	1.403814	1.455812
2	6	0	-2.661614	2.464476	1.230959
3	6	0	-1.553774	2.503815	0.206848
4	6	0	-0.139071	2.430665	0.815285
5	6	0	0.173152	1.956820	2.080034
6	6	0	-0.586862	1.022928	3.034476
7	6	0	0.413386	-0.117039	3.267391
8	6	0	0.365396	-1.494464	3.063596
9	6	0	-0.540729	-2.300482	2.141709
10	6	0	-1.618793	-1.874106	1.355325
11	6	0	-2.700328	-0.955422	1.813934
12	6	0	-3.247504	0.081007	0.764394
13	6	0	-2.272425	0.157884	-0.389200
14	6	0	-1.566077	1.293919	-0.713198
15	6	0	-0.533174	1.256512	-1.721861
16	6	0	0.706778	2.011931	-1.517921
17	6	0	0.948000	2.532186	-0.160975
18	6	0	2.275151	2.574274	0.300605
19	6	0	2.587630	2.195667	1.660410
20	6	0	1.544319	1.758986	2.454862
21	6	0	1.691692	0.555805	3.252525
22	6	0	2.906591	-0.112065	3.262967
23	6	0	2.884037	-1.547196	3.202591
24	6	0	1.651184	-2.199949	3.079132
25	6	0	1.577905	-3.364453	2.234243
26	6	0	0.262844	-3.390547	1.646727
27	6	0	0.089108	-3.916244	0.365625
28	6	0	-0.838397	-3.276058	-0.524222
29	6	0	-1.632706	-2.218972	-0.060975
30	6	0	-1.891597	-1.103558	-0.974220
31	6	0	-1.201736	-1.066918	-2.185134
32	6	0	-0.529851	0.153702	-2.585342
33	6	0	0.630990	-0.212650	-3.353662
34	6	0	1.784104	0.548445	-3.239801
35	6	0	1.819529	1.672583	-2.318076
36	6	0	3.177182	1.752744	-1.813080
37	6	0	3.398877	2.185222	-0.515137
38	6	0	4.383511	1.518450	0.323009
39	6	0	3.862836	1.504572	1.673616
40	6	0	4.025919	0.370673	2.472112
41	6	0	4.726219	-0.787334	1.953158
42	6	0	4.032218	-1.978634	2.423534
43	6	0	3.936342	-3.096981	1.597557
44	6	0	2.685368	-3.828452	1.526922
45	6	0	2.515028	-4.307645	0.164930
46	6	0	1.241173	-4.361744	-0.398713
47	6	0	1.038416	-3.950713	-1.776096
48	6	0	-0.250774	-3.289533	-1.850539
49	6	0	-0.413258	-2.178164	-2.665769
50	6	0	0.709248	-1.660071	-3.420535
51	6	0	1.947625	-2.295888	-3.365954
52	6	0	3.160326	-1.500903	-3.279645
53	6	0	3.079091	-0.105940	-3.232975
54	6	0	3.946697	0.644773	-2.347013
55	6	0	4.869297	-0.021667	-1.536657
56	6	0	4.928796	-1.472806	-1.556729
57	6	0	4.091699	-2.196558	-2.407960
58	6	0	3.440844	-3.408028	-1.933988
59	6	0	2.117166	-3.471897	-2.526236
60	6	0	3.639621	-3.832238	-0.616710
61	6	0	4.516642	-3.079915	0.268394
62	6	0	5.157241	-1.927986	-0.194798
63	6	0	5.263019	-0.760326	0.661902
64	6	0	5.086996	0.419600	-0.170464
65	6	0	-4.588549	-0.461506	0.234695
66	6	0	-5.584494	0.397911	-0.237128
67	6	0	-6.749538	-0.177127	-0.736579
68	6	0	-6.880604	-1.566345	-0.752549
69	6	0	-5.829624	-2.355700	-0.268193
70	6	0	-1.718563	3.828692	-0.558833
71	6	0	-2.546588	3.927278	-1.681409
72	6	0	-2.680663	5.176287	-2.278573
73	6	0	-1.996733	6.270728	-1.745709
74	6	0	-1.197627	6.086533	-0.611068
75	7	0	-1.082509	4.875969	-0.036471
76	8	0	-1.624074	1.179840	3.626178
77	8	0	-3.184081	-1.045350	2.921414
78	7	0	-4.709862	-1.788203	0.210878
79	6	0	-5.839795	-3.887895	-0.241643
80	6	0	-0.381712	7.196414	0.060413
81	6	0	-5.593262	-4.354102	1.211250
82	6	0	-4.691142	-4.396056	-1.143161
83	6	0	-7.170307	-4.473760	-0.741954
84	6	0	-0.811796	7.296191	1.541111
85	6	0	-0.578173	8.562502	-0.616823
86	6	0	1.111885	6.802043	-0.012341
87	1	0	-4.179296	1.420910	2.241205
88	1	0	-2.781590	3.369070	1.819837
89	1	0	-5.443883	1.473484	-0.209612
90	1	0	-7.553245	0.450963	-1.112083
91	1	0	-7.786640	-2.018673	-1.137019
92	1	0	-3.055911	3.052098	-2.071578
93	1	0	-3.308797	5.299926	-3.156930
94	1	0	-2.088774	7.244683	-2.210911
95	1	0	-5.534353	-5.448295	1.251064
96	1	0	-6.408675	-4.032492	1.869937
97	1	0	-4.660438	-3.936914	1.599838
98	1	0	-4.655940	-5.491910	-1.126650
99	1	0	-3.727121	-4.013220	-0.798119
100	1	0	-4.834690	-4.075881	-2.182219

101	1	0	-7.133107	-5.567334	-0.690560
102	1	0	-7.371020	-4.200240	-1.784677
103	1	0	-8.016494	-4.142298	-0.128570
104	1	0	-0.205509	8.047289	2.061067
105	1	0	-0.684758	6.333831	2.044219
106	1	0	-1.864787	7.590918	1.625458
107	1	0	0.019781	9.320218	-0.098780
108	1	0	-1.625078	8.886666	-0.582045
109	1	0	-0.255731	8.548215	-1.664535
110	1	0	1.726671	7.550859	0.501197
111	1	0	1.450357	6.739345	-1.053597
112	1	0	1.278381	5.828520	0.457430

The total electronic energy was calculated to be –3400.3174896 Hartree.

Table S4. Optimized structure of **2** (B3LYP-D3/6-31G(d))

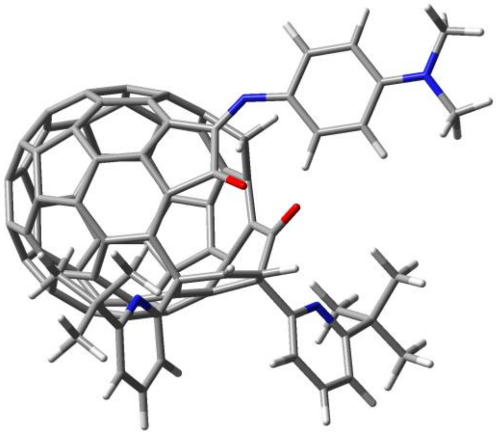

Standard orientation:

Center Number	Atomic Number	Atomic Type	Coordinates (Angstroms) X	Y	Z
1	6	0	2.444140	1.790935	1.402512
2	6	0	1.651160	2.766728	0.969142
3	6	0	0.144459	2.708447	0.983781
4	6	0	-0.482095	2.508870	-0.415311
5	6	0	0.215238	2.160715	-1.569970
6	6	0	1.657429	1.880864	-1.938352
7	6	0	1.609544	0.704925	-2.942010
8	6	0	1.597390	-2.194298	-1.626788
9	6	0	1.305179	-2.632058	-0.195603
10	6	0	1.414518	-1.858133	0.955040
11	6	0	2.296111	-0.665874	0.960057
12	6	0	1.946481	0.484596	1.958999
13	6	0	0.446597	0.475461	2.169019
14	6	0	-0.372057	1.514948	1.785357
15	6	0	-1.807417	1.377163	1.870881
16	6	0	-2.641206	1.931975	0.807576
17	6	0	-1.943276	2.386531	-0.406341
18	6	0	-2.617610	2.214073	-1.628883
19	6	0	-1.877554	1.766401	-2.773695
20	6	0	-0.522177	1.598670	-2.669731
21	6	0	0.188246	0.476558	-3.237175
22	6	0	-0.584991	-0.624076	-3.635865
23	6	0	-0.467619	-2.096362	-3.303477
24	6	0	0.371298	-2.726761	-2.380853
25	6	0	-0.155294	-3.821716	-1.591780
26	6	0	0.376324	-3.708037	-0.249392
27	6	0	-0.399729	-4.046196	0.857353
28	6	0	-0.395403	-3.163663	1.998633
29	6	0	0.422235	-2.018861	2.005941
30	6	0	-0.124573	-0.787945	2.580660
31	6	0	-1.451621	-0.780582	3.005786
32	6	0	-2.306754	0.336236	2.663963
33	6	0	-3.647054	-0.166030	2.514640
34	6	0	-4.470162	0.389813	1.549585
35	6	0	-3.967183	1.452283	0.696930
36	6	0	-4.619239	1.288697	-0.591050
37	6	0	-3.935952	1.640573	-1.740941
38	6	0	-3.965785	0.766290	-2.907340
39	6	0	-2.647340	0.802873	-3.495035
40	6	0	-2.002438	-0.383119	-3.870607
41	6	0	-2.740180	-1.616425	-3.812770
42	6	0	-1.808763	-2.665748	-3.480880
43	6	0	-2.278127	-3.767350	-2.757384
44	6	0	-1.426579	-4.359640	-1.769704
45	6	0	-2.236471	-4.733364	-0.620344
46	6	0	-1.734346	-4.576432	0.672206
47	6	0	-2.575177	-4.027995	1.717950
48	6	0	-1.743423	-3.158206	2.531708
49	6	0	-2.268958	-1.972644	3.028312
50	6	0	-3.635237	-1.601254	2.737952
51	6	0	-4.442820	-2.434659	1.964438
52	6	0	-5.315032	-1.855402	0.957130
53	6	0	-5.346147	-0.470313	0.769800
54	6	0	-5.427312	0.083830	-0.564080
55	6	0	-5.446247	-0.767499	-1.676738
56	6	0	-5.365472	-2.205747	-1.485192
57	6	0	-5.313367	-2.739934	-0.195611
58	6	0	-4.426956	-3.854991	0.094230
59	6	0	-3.897701	-3.672900	1.435495
60	6	0	-3.609069	-4.375119	-0.915412
61	6	0	-3.640187	-3.793919	-2.249277
62	6	0	-4.513566	-2.746605	-2.531302
63	6	0	-4.064882	-1.645687	-3.360591
64	6	0	-4.679261	-0.427755	-2.858226
65	6	0	2.635190	0.182735	3.302312
66	6	0	2.726668	1.165354	4.294297
67	6	0	3.326651	0.813248	5.497928
68	6	0	3.807307	-0.486826	5.674367
69	6	0	3.675299	-1.409187	4.630411
70	6	0	-0.337346	4.046774	1.575867
71	6	0	-0.453986	4.240306	2.956530
72	6	0	-0.841068	5.499098	3.403531
73	6	0	-1.095028	6.511447	2.475631
74	6	0	-0.944273	6.235532	1.111647
75	7	0	-0.562875	5.016310	0.691704
76	8	0	2.646362	2.521813	-1.640830
77	8	0	3.234658	-0.551395	0.188432
78	7	0	3.095279	-1.055551	3.468791
79	6	0	4.141742	-2.867228	4.696680
80	6	0	-1.207748	7.244729	-0.011054
81	6	0	5.119230	-3.123527	3.527413
82	6	0	2.901832	-3.776866	4.533850
83	6	0	4.841585	-3.199126	6.024376
84	6	0	0.069134	7.367163	-0.872425
85	6	0	-1.592946	8.633487	0.524362
86	6	0	-2.360472	6.696860	-0.884366
87	6	0	6.749441	-0.110532	-3.522440
88	6	0	5.969398	-0.561826	-4.619166
89	6	0	4.591473	-0.476572	-4.580605

90	6	0	3.900443	0.099647	-3.487981
91	6	0	4.679066	0.534225	-2.387737
92	6	0	6.055311	0.416839	-2.400966
93	7	0	8.122639	-0.193616	-3.538164
94	7	0	2.528327	0.073076	-3.596972
95	6	0	8.892545	0.214953	-2.373419
96	6	0	8.803996	-0.806301	-4.667140
97	1	0	3.521642	1.888870	1.322208
98	1	0	2.076130	3.661066	0.528237
99	1	0	2.530150	-2.607720	-2.030790
100	1	0	1.699303	-1.120131	-1.678144
101	1	0	2.341097	2.163708	4.113901
102	1	0	3.420699	1.542460	6.298531
103	1	0	4.273597	-0.766856	6.611297
104	1	0	-0.257743	3.426566	3.647228
105	1	0	-0.950750	5.693881	4.467286
106	1	0	-1.404765	7.492005	2.816434
107	1	0	5.423595	-4.176878	3.514357
108	1	0	6.022164	-2.509449	3.629582
109	1	0	4.648781	-2.881330	2.570708
110	1	0	3.203837	-4.831061	4.538084
111	1	0	2.384643	-3.565770	3.593993
112	1	0	2.190897	-3.623132	5.354649
113	1	0	5.163557	-4.246215	6.019546
114	1	0	4.173085	-3.064290	6.882861
115	1	0	5.732536	-2.579086	6.179418
116	1	0	-0.108840	8.043006	-1.717445
117	1	0	0.365647	6.389280	-1.261097
118	1	0	0.902816	7.769660	-0.284376
119	1	0	-1.760250	9.318160	-0.314340
120	1	0	-0.800108	9.062294	1.148806
121	1	0	-2.517309	8.602310	1.113226
122	1	0	-2.545545	7.368737	-1.730941
123	1	0	-3.287654	6.615446	-0.304348
124	1	0	-2.112608	5.704200	-1.270985
125	1	0	6.442069	-0.997172	-5.491020
126	1	0	3.997760	-0.848322	-5.410336
127	1	0	4.195005	0.922741	-1.505931
128	1	0	6.601469	0.735306	-1.521452
129	1	0	8.714953	1.269898	-2.128196
130	1	0	9.956134	0.093648	-2.584115
131	1	0	8.647745	-0.387784	-1.487273
132	1	0	8.518262	-1.860057	-4.796951
133	1	0	9.881854	-0.764682	-4.503786
134	1	0	8.584473	-0.275537	-5.602767

The total electronic energy was calculated to be –3821.9488636 Hartree.

Table S5. Optimized structure of **II** (B3LYP-D3/6-31G(d))

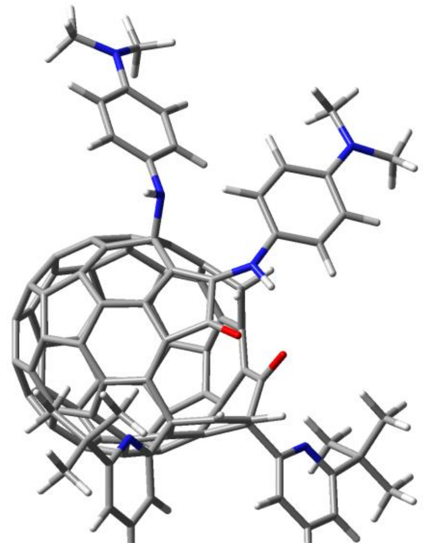

Standard orientation:

Center Number	Atomic Number	Atomic Type	Coordinates (Angstroms) X	Y	Z
1	8	0	1.329735	0.990609	3.995345
2	8	0	1.015380	3.156809	1.436259
3	7	0	3.311530	4.405323	-0.610163
4	7	0	4.616742	-2.293844	2.869672
5	7	0	-3.702487	-1.003822	2.333592
6	7	0	-1.201501	1.652040	3.458941
7	6	0	-2.816858	-0.166351	0.087777
8	6	0	-3.000525	-2.464550	-0.513503
9	6	0	3.113415	0.239640	-2.684010
10	6	0	3.453092	-1.347283	-0.833888
11	6	0	3.216005	1.049930	-0.399942
12	6	0	2.791984	1.242309	-1.767854
13	6	0	3.065707	2.250501	0.513153
14	6	0	3.468641	-1.080595	-2.206653
15	6	0	-2.385929	1.076602	-0.350740
16	6	0	-3.345569	-0.811851	-2.285747
17	6	0	2.873617	-2.604719	-0.356912
18	6	0	-3.289164	-1.107277	-0.922857
19	6	0	2.362011	-1.328909	-4.263843
20	6	0	-2.856696	0.458531	-2.731680
21	6	0	2.166049	-2.543756	0.918648
22	6	0	0.720152	0.273622	3.226285
23	6	0	3.506050	-0.214881	0.062545
24	6	0	-2.362053	-2.411326	0.757589
25	6	0	-2.373799	1.345322	-1.778740
26	6	0	4.796580	-1.390615	1.907772
27	6	0	6.072704	4.399571	-0.421020
28	6	0	3.425054	1.938675	1.940454
29	6	0	1.229702	-1.867607	-4.876777
30	6	0	-1.260364	-0.623888	2.227787
31	6	0	1.571617	2.711851	0.444122
32	6	0	-0.153037	-2.819714	1.701893
33	6	0	-1.334707	-3.297767	1.006167
34	6	0	1.013497	-3.341615	1.054343
35	6	0	3.565806	-0.537587	1.555628
36	6	0	3.014996	-2.039471	-3.183177
37	6	0	7.108044	-1.957601	1.707345
38	6	0	0.866897	2.522207	-0.848844
39	6	0	-2.664385	-3.441867	-1.471876
40	6	0	-1.566032	-4.339511	-1.210065
41	6	0	-1.462189	2.125237	0.283304
42	6	0	-2.342437	2.467490	3.308944
43	6	0	-2.978074	-1.807291	-3.281273
44	6	0	-0.797113	0.461805	2.960573
45	6	0	-2.521711	-0.996437	1.406142
46	6	0	3.639859	0.709297	2.400109
47	6	0	4.011978	5.428355	-1.132974
48	6	0	-3.633003	1.972303	3.125795
49	6	0	2.417823	0.081311	-3.939382
50	6	0	-2.623202	-3.090625	-2.879195
51	6	0	0.720686	-3.154634	-4.437713

52	6	0	1.365461	-3.846407	-3.406189	106	6	0	-9.509410	-0.908344	-0.578443
53	6	0	0.559690	-4.257437	0.040739	107	1	0	-3.487479	-1.673039	3.069289
54	6	0	1.614225	2.073314	-2.018728	108	1	0	-0.405071	2.128879	3.882417
55	6	0	2.522885	-3.260899	-2.747781	109	1	0	7.157253	4.404304	-0.348424
56	6	0	3.945015	3.396620	-0.015566	110	1	0	3.445725	2.785089	2.618664
57	6	0	-1.200497	2.131627	-2.082524	111	1	0	8.088087	-1.828265	1.255320
58	6	0	5.337960	3.345268	0.108760	112	1	0	-0.910423	1.765010	1.137061
59	6	0	-4.535007	4.245250	2.982814	113	1	0	-2.030468	3.000655	0.618971
60	6	0	-0.520582	2.449760	-0.872895	114	1	0	3.835843	0.546455	3.454256
61	6	0	2.460457	-3.550466	-1.328553	115	1	0	-3.794046	0.899951	3.089937
62	6	0	-4.704446	2.843108	2.956280	116	1	0	5.812955	2.506039	0.606904
63	6	0	1.338483	0.907392	-4.218272	117	1	0	-5.685031	2.407819	2.807691
64	6	0	1.138873	-0.915926	2.430310	118	1	0	-1.143993	4.257736	3.434388
65	6	0	-2.149887	3.856729	3.335162	119	1	0	7.743498	-3.541457	3.019227
66	6	0	5.291555	-4.092425	4.349562	120	1	0	5.976067	6.276780	-1.471381
67	6	0	-2.156385	0.273225	-3.990903	121	1	0	6.143722	-0.424720	0.518075
68	6	0	2.277493	-1.358429	1.769766	122	1	0	-3.015620	5.792135	3.180890
69	6	0	-0.123487	-1.542784	2.166281	123	1	0	3.339618	-4.468154	3.438213
70	6	0	-0.509337	1.950135	-3.276950	124	1	0	3.890504	-5.755473	4.538848
71	6	0	0.931452	1.893559	-3.235994	125	1	0	4.599504	-5.601319	2.918077
72	6	0	1.287820	-4.379216	-1.131713	126	1	0	5.467300	-2.675511	6.012679
73	6	0	3.160091	6.507026	-1.810328	127	1	0	4.404028	-4.058514	6.341766
74	6	0	-0.895279	-4.240438	0.009219	128	1	0	3.852344	-2.739594	5.280973
75	6	0	6.915280	-2.919924	2.700822	129	1	0	6.931859	-5.497079	3.958611
76	6	0	-0.806621	-4.503881	-2.437568	130	1	0	6.206290	-5.636740	5.564415
77	6	0	5.644212	-3.064233	3.268903	131	1	0	7.299111	-4.291281	5.212660
78	6	0	-0.730425	-3.095853	-4.467880	132	1	0	4.573750	8.167587	-1.566276
79	6	0	5.408313	5.454666	-1.052430	133	1	0	3.357568	8.411314	-2.826843
80	6	0	-1.115759	-1.765545	-4.908780	134	1	0	4.717735	7.322369	-3.123837
81	6	0	6.035890	-1.173522	1.296121	135	1	0	1.544798	6.249734	-0.361809
82	6	0	-0.996352	1.003764	-4.258477	136	1	0	1.490936	7.792751	-1.248763
83	6	0	-1.476640	-3.748911	-3.481837	137	1	0	2.674008	7.553324	0.053527
84	6	0	-3.218081	4.728215	3.172776	138	1	0	3.069782	5.450254	-3.730626
85	6	0	-2.222715	-1.135139	-4.326116	139	1	0	1.731600	6.584743	-3.457871
86	7	0	-5.614063	5.111186	2.834615	140	1	0	1.766458	5.021370	-2.609451
87	6	0	0.095866	-1.007697	-5.169773	141	1	0	-5.245644	0.744568	1.043245
88	6	0	0.152382	0.353530	-4.851494	142	1	0	-5.103790	-3.315738	2.408601
89	6	0	0.592551	-4.536636	-2.398145	143	1	0	-7.364663	-3.690951	1.589381
90	6	0	4.210833	-5.037341	3.774538	144	1	0	-7.485271	0.386924	0.158474
91	6	0	4.716463	-3.342661	5.572115	145	1	0	-7.614084	5.379845	2.308559
92	6	0	6.507950	-4.922328	4.790485	146	1	0	-7.276138	3.839106	3.098826
93	6	0	4.009115	7.664330	-2.360187	147	1	0	-6.812983	4.076946	1.398435
94	6	0	2.153529	7.058441	-0.775258	148	1	0	-6.306793	7.051278	2.512493
95	6	0	2.383170	5.847530	-2.973493	149	1	0	-4.766906	6.706378	1.690090
96	6	0	-5.006733	-1.252579	1.803317	150	1	0	-4.816072	6.964105	3.449697
97	6	0	-5.713104	-0.226801	1.162233	151	1	0	-10.475761	-3.220974	-0.116948
98	6	0	-5.636226	-2.494023	1.934449	152	1	0	-9.493380	-3.693911	1.268412
99	6	0	-6.927717	-2.708267	1.460609	153	1	0	-8.849516	-3.895973	-0.377842
100	6	0	-6.992298	-0.436392	0.661051	154	1	0	-10.516044	-1.223955	-0.860697
101	6	0	-7.645101	-1.681816	0.808399	155	1	0	-8.912189	-0.788274	-1.497527
102	6	0	-6.885022	4.570363	2.381942	156	1	0	-9.597774	0.074501	-0.100870
103	6	0	-5.355849	6.522509	2.603818						
104	7	0	-8.944056	-1.882338	0.340634						
105	6	0	-9.455052	-3.241237	0.270802						

The total electronic energy was calculated to be –4243.5522253 Hartree.

Table S6. Optimized structure of **3** (B3LYP-D3/6-31G(d))

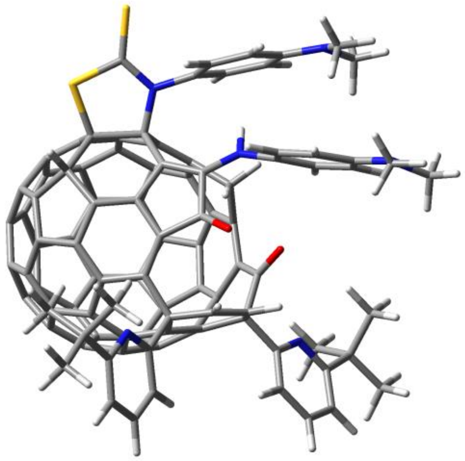

Standard orientation:

Center Number	Atomic Number	Atomic Type	Coordinates (Angstroms) X	Y	Z
1	8	0	-2.407169	1.082068	2.330047
2	8	0	-1.996796	2.244345	-1.075275
3	7	0	-0.206221	4.824182	-2.228781
4	7	0	1.506080	2.040125	4.686353
5	7	0	-2.065059	-4.204719	0.052357
6	7	0	-3.371660	-0.830220	0.348499
7	6	0	-0.454861	-2.894316	-1.332652
8	6	0	1.238552	-4.316685	-0.502087
9	6	0	3.479546	2.330488	-1.265484
10	6	0	3.196718	1.727267	1.100874
11	6	0	1.397564	2.846927	-0.125846
12	6	0	2.116573	2.614115	-1.358655
13	6	0	-0.015487	3.382357	-0.265407
14	6	0	4.040491	1.903962	-0.001306
15	6	0	-0.700685	-1.832328	-2.196189
16	6	0	1.402230	-3.664805	-2.844962
17	6	0	3.376428	0.547274	1.945711
18	6	0	0.636659	-3.788360	-1.674714
19	6	0	5.249791	0.849861	-1.715306
20	6	0	1.145706	-2.566839	-3.722008
21	6	0	2.151506	-0.042667	2.477155
22	6	0	-1.713568	0.187394	1.898175
23	6	0	1.904180	2.374021	1.065528
24	6	0	0.364711	-4.115467	0.706152
25	6	0	0.139607	-1.679450	-3.369379
26	6	0	1.710428	2.739871	3.572665
27	6	0	0.441717	7.125263	-0.832782
28	6	0	-0.745789	3.454191	1.048282
29	6	0	5.580407	-0.393602	-2.259087
30	6	0	-1.250279	-1.986050	1.017883
31	6	0	-0.797826	2.422840	-1.216719
32	6	0	0.920424	-2.168177	2.281815
33	6	0	1.201461	-3.599775	1.948727
34	6	0	2.104582	-1.442094	2.583391
35	6	0	1.024253	2.199409	2.305752
36	6	0	5.136340	1.008930	-0.280214
37	6	0	2.991345	4.376297	4.749833
38	6	0	-0.012502	1.700576	-2.244972
39	6	0	2.605314	-4.544522	-0.459799
40	6	0	3.386399	-4.115530	0.719031
41	6	0	-1.424056	-0.487012	-2.017057
42	6	0	-4.459471	0.048196	0.612217
43	6	0	2.822801	-3.902086	-2.821940
44	6	0	-2.225529	-0.999437	1.050266
45	6	0	-0.928265	-3.221886	0.138757
46	6	0	-0.290377	2.924845	2.179203
47	6	0	-0.142445	5.998477	-2.882300
48	6	0	-4.944042	0.854695	-0.417738
49	6	0	4.212173	1.659834	-2.316877
50	6	0	3.417550	-4.294817	-1.623322
51	6	0	5.834989	-1.517283	-1.378008
52	6	0	5.755097	-1.354712	0.008887
53	6	0	3.219670	-2.301366	2.309008
54	6	0	1.369090	2.079578	-2.500487
55	6	0	5.364858	-0.067577	0.565549
56	6	0	0.042686	4.773389	-0.921213
57	6	0	0.320841	-0.270989	-3.628815
58	6	0	0.375717	5.905245	-0.169204
59	6	0	-6.771937	1.647362	0.994679
60	6	0	-0.470807	0.478778	-2.720097
61	6	0	4.486111	-0.292731	1.695272
62	6	0	-6.080539	1.636589	-0.237823
63	6	0	3.523620	1.202460	-3.431294
64	6	0	-0.254401	-0.079061	2.122983
65	6	0	-5.125561	0.052343	1.839211
66	6	0	1.774808	1.527395	7.040206
67	6	0	2.412885	-2.080494	-4.241781
68	6	0	0.883325	0.666187	2.397883
69	6	0	-0.164388	-1.486862	1.859183
70	6	0	1.536439	0.212599	-4.107648
71	6	0	2.087197	1.398729	-3.500142
72	6	0	4.409763	-1.731696	1.874961
73	6	0	-0.428835	5.913981	-4.385391
74	6	0	2.719846	-3.540997	1.769966
75	6	0	2.795272	3.631158	5.914899
76	6	0	4.628883	-3.543607	0.239034
77	6	0	2.040616	2.454298	5.848942
78	6	0	5.271530	-2.705661	-1.997049
79	6	0	0.180855	7.179053	-2.204097
80	6	0	4.660428	-2.309262	-3.256891
81	6	0	2.444511	3.930789	3.551565
82	6	0	2.610778	-0.709311	-4.416338
83	6	0	4.667742	-3.684417	-1.202743
84	6	0	-6.247645	0.846497	2.037498
85	6	0	3.458335	-2.901352	-3.663177
86	7	0	-7.928491	2.403282	1.173137
87	6	0	4.857370	-0.881494	-3.421269
88	6	0	3.851638	-0.094966	-3.997498
89	6	0	5.157380	-2.385713	0.829531
90	6	0	2.413786	0.154375	6.725141
91	6	0	0.247943	1.354447	7.199734
92	6	0	2.363144	2.071459	8.351935
93	6	0	-0.419484	7.294493	-5.062069
94	6	0	-1.813129	5.257819	-4.587489
95	6	0	0.657614	5.023332	-5.031595

96	6	0	-3.368751	-3.770148	-0.357006
97	6	0	-4.388964	-3.676822	0.597961
98	6	0	-3.644305	-3.405667	-1.674666
99	6	0	-4.878969	-2.848531	-2.011015
100	6	0	-5.629069	-3.159415	0.267342
101	6	0	-5.901189	-2.690987	-1.043814
102	6	0	-8.272457	3.399499	0.172092
103	6	0	-8.434506	2.600312	2.521983
104	7	0	-7.106773	-2.093811	-1.357679
105	6	0	-7.220842	-1.364850	-2.611203
106	6	0	-8.022908	-1.741174	-0.278997
107	6	0	-1.904694	-5.495432	0.456065
108	16	0	-3.014316	-6.730762	0.378155
109	16	0	-0.329734	-5.771646	1.170961
110	1	0	-3.539242	-1.487979	-0.400313
111	1	0	0.695348	8.032282	-0.290083
112	1	0	-1.726263	3.917104	1.017425
113	1	0	3.573917	5.293464	4.780203
114	1	0	-1.561167	-0.197299	-0.985533
115	1	0	-2.422744	-0.511133	-2.472964
116	1	0	0.946931	-4.245000	2.799958
117	1	0	-0.892853	2.956947	3.079488
118	1	0	-4.398492	0.901764	-1.354738
119	1	0	0.567943	5.819330	0.895491
120	1	0	-6.409048	2.259383	-1.060562
121	1	0	-4.752864	-0.568689	2.647364
122	1	0	3.228088	3.965948	6.849959
123	1	0	0.230683	8.126822	-2.726395
124	1	0	2.586305	4.475166	2.623268
125	1	0	-6.720444	0.832163	3.011711
126	1	0	2.019015	-0.249192	5.788281
127	1	0	2.201813	-0.556834	7.532596
128	1	0	3.502455	0.242076	6.625571
129	1	0	-0.231568	2.310212	7.443583
130	1	0	0.030646	0.648136	8.009847
131	1	0	-0.194351	0.975968	6.274384
132	1	0	3.453421	2.173770	8.298702
133	1	0	2.137541	1.381081	9.172132
134	1	0	1.936794	3.047258	8.613389
135	1	0	-1.175929	7.962029	-4.632461
136	1	0	-0.642449	7.183807	-6.128935
137	1	0	0.558649	7.782959	-4.980145
138	1	0	-1.848617	4.276973	-4.106088
139	1	0	-2.018616	5.134556	-5.657502
140	1	0	-2.608024	5.878664	-4.156900
141	1	0	1.652631	5.468730	-4.912504
142	1	0	0.462855	4.907939	-6.104603
143	1	0	0.672948	4.031826	-4.570820
144	1	0	-4.184364	-3.984480	1.617490
145	1	0	-2.884120	-3.522684	-2.437483
146	1	0	-5.039783	-2.541957	-3.036726
147	1	0	-6.373942	-3.068761	1.046704
148	1	0	-9.197505	3.899567	0.466277
149	1	0	-8.450554	2.927992	-0.801744
150	1	0	-7.489894	4.164692	0.041443
151	1	0	-9.331120	3.222175	2.481662
152	1	0	-7.702879	3.087556	3.186786
153	1	0	-8.720292	1.643924	2.975621
154	1	0	-8.219160	-0.930012	-2.682432
155	1	0	-7.091457	-2.033504	-3.470217
156	1	0	-6.482917	-0.550975	-2.685686
157	1	0	-8.906739	-1.264302	-0.706266
158	1	0	-7.570255	-1.049555	0.444366
159	1	0	-8.358920	-2.639198	0.252352

The total electronic energy was calculated to be –5078.064729 Hartree.

5.2. Rotation of Phenylene Rings in 3

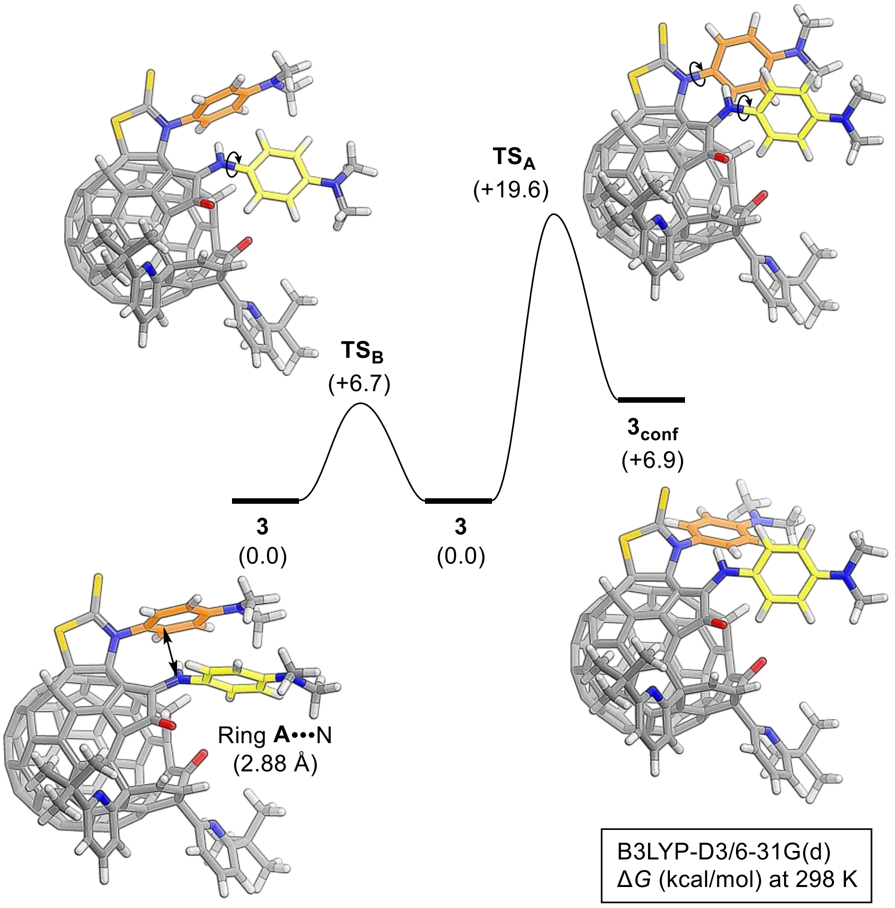

Figure S11. Rotational barriers of phenylene rings in **3** (B3LYP-D3/6-31G(d)).

Table S7. Optimized structure of $\mathbf{3_{conf}}$ (B3LYP-D3/6-31G(d))

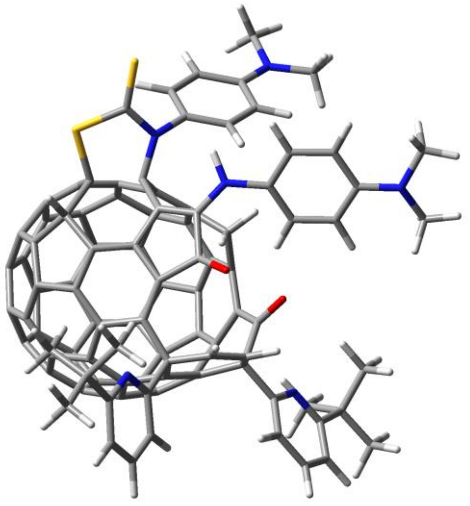

Standard orientation:

Center Number	Atomic Number	Atomic Type	Coordinates (Angstroms) X	Y	Z
1	8	0	-0.042490	2.048060	3.354394
2	8	0	-0.266671	3.474028	-0.209994
3	7	0	2.179834	4.445027	-2.249331
4	7	0	4.217719	0.187463	3.705484
5	7	0	-3.810902	-1.846591	1.688153
6	7	0	-2.812403	1.015141	2.884353
7	6	0	-2.382896	-1.628625	-0.287259
8	6	0	-1.833959	-3.921000	-0.052094
9	6	0	3.444708	0.174292	-2.554366
10	6	0	3.827410	-0.519884	-0.227069
11	6	0	2.784572	1.638975	-0.728546
12	6	0	2.647072	1.229203	-2.109872
13	6	0	2.090460	2.931924	-0.338072
14	6	0	4.076930	-0.702744	-1.592445
15	6	0	-2.264993	-0.494759	-1.074515
16	6	0	-2.233326	-3.074066	-2.320796
17	6	0	3.578548	-1.695955	0.605696
18	6	0	-2.354743	-2.925333	-0.931841
19	6	0	3.581653	-1.973602	-3.504334
20	6	0	-2.098725	-1.905589	-3.131543
21	6	0	2.584693	-1.523679	1.658928
22	6	0	-0.245004	0.991404	2.796024
23	6	0	3.310342	0.763655	0.197196
24	6	0	-1.813174	-3.418700	1.373908
25	6	0	-2.094804	-0.670054	-2.499690
26	6	0	4.361428	0.766351	2.515277
27	6	0	4.480335	5.716685	-1.380899
28	6	0	2.100791	3.185090	1.145495
29	6	0	2.839401	-3.060929	-3.972828
30	6	0	-1.505611	-0.888441	2.059350
31	6	0	0.601372	2.839626	-0.788102
32	6	0	0.404121	-2.462460	2.260772
33	6	0	-0.396830	-3.705360	2.047260
34	6	0	1.776828	-2.631873	1.957646
35	6	0	3.068825	0.941097	1.699039
36	6	0	4.178282	-2.014490	-2.185219
37	6	0	6.696792	1.104051	2.880262
38	6	0	0.280472	1.923198	-1.900245
39	6	0	-0.986203	-4.894376	-0.571785
40	6	0	0.296428	-5.207633	0.099022
41	6	0	-1.881136	0.962283	-0.761407
42	6	0	-3.153994	2.387512	2.853747
43	6	0	-1.394758	-4.106383	-2.871340
44	6	0	-1.651804	0.392642	2.534678
45	6	0	-2.367980	-1.856248	1.250159
46	6	0	2.519570	2.300642	2.043896
47	6	0	2.717940	5.422839	-2.999597
48	6	0	-2.462409	3.323651	2.079465
49	6	0	3.115722	-0.620405	-3.718439
50	6	0	-0.740165	-4.970394	-1.993412
51	6	0	2.695065	-4.242351	-3.143917
52	6	0	3.299958	-4.294055	-1.884619
53	6	0	1.920794	-3.927571	1.362207
54	6	0	1.349616	1.437595	-2.758432
55	6	0	4.028478	-3.137153	-1.383495
56	6	0	2.752957	4.094322	-1.098827
57	6	0	-1.237650	0.378360	-2.992199
58	6	0	3.915849	4.701998	-0.614847
59	6	0	-3.996899	5.101734	2.762258
60	6	0	-0.955023	1.291094	-1.940760
61	6	0	3.744025	-2.978683	0.027733
62	6	0	-2.869204	4.649606	2.039684
63	6	0	1.914818	-0.390123	-4.374577
64	6	0	0.739496	-0.072340	2.346346
65	6	0	-4.287495	2.810316	3.556415
66	6	0	4.987508	-0.693474	5.828418
67	6	0	-1.154061	-2.185517	-4.198679
68	6	0	2.084707	-0.199656	2.017379
69	6	0	-0.108393	-1.220903	2.236310
70	6	0	-0.319053	0.121191	-4.008555
71	6	0	1.021231	0.631498	-3.861988
72	6	0	2.905954	-4.104570	0.401929
73	6	0	1.971260	5.719275	-4.304681
74	6	0	0.611534	-4.525430	1.243810
75	6	0	6.550822	0.479612	4.120971
76	6	0	1.303651	-5.400691	-0.928190
77	6	0	5.284030	0.027511	4.508965
78	6	0	1.341500	-4.744617	-3.311184
79	6	0	3.879829	6.085314	-2.586597
80	6	0	0.649009	-3.861893	-4.237560
81	6	0	5.588259	1.255005	2.054184
82	6	0	-0.266064	-1.192631	-4.618649
83	6	0	0.659332	-5.292002	-2.220149
84	6	0	-4.708669	4.134927	3.506761
85	6	0	-0.698129	-3.550143	-4.020861
86	7	0	-4.386662	6.440250	2.741541
87	6	0	1.576967	-2.826473	-4.652706
88	6	0	1.125660	-1.514785	-4.848387
89	6	0	2.602729	-4.892263	-0.767606
90	6	0	3.884390	0.086546	6.578107
91	6	0	6.228647	-0.807405	6.728187
92	6	0	4.470198	-2.111214	5.490158
93	6	0	2.596748	6.887188	-5.085087
94	6	0	0.503946	6.063219	-3.963163
95	6	0	2.006298	4.445487	-5.180538

96	6	0	-4.818640	-1.268457	0.818012
97	6	0	-5.010189	0.105104	0.681482
98	6	0	-5.584694	-2.127265	0.020332
99	6	0	-6.513671	-1.635901	-0.882756
100	6	0	-5.930889	0.612396	-0.229650
101	6	0	-6.707552	-0.241961	-1.044204
102	6	0	-3.795809	7.324172	1.751076
103	6	0	-5.690530	6.796768	3.272198
104	7	0	-7.610139	0.258823	-1.964886
105	6	0	-8.510225	-0.650493	-2.654162
106	6	0	-7.889918	1.684267	-1.989765
107	6	0	-4.210589	-2.875203	2.510946
108	16	0	-5.605485	-2.952641	3.396227
109	16	0	-3.071773	-4.221855	2.489796
110	1	0	-3.583595	0.378499	3.049252
111	1	0	5.383487	6.219763	-1.045181
112	1	0	1.671711	4.129298	1.463281
113	1	0	7.671829	1.463669	2.561680
114	1	0	-1.363723	1.074909	0.187348
115	1	0	-2.769787	1.603756	-0.716690
116	1	0	-0.589766	-4.198512	3.008258
117	1	0	2.435693	2.512666	3.103639
118	1	0	-1.598304	3.036637	1.498147
119	1	0	4.349933	4.385633	0.328104
120	1	0	-2.296862	5.325332	1.416392
121	1	0	-4.853208	2.094172	4.148500
122	1	0	7.411762	0.350353	4.765766
123	1	0	4.314206	6.874790	-3.187706
124	1	0	5.663815	1.725811	1.079169
125	1	0	-5.598172	4.407864	4.061027
126	1	0	4.229411	1.091825	6.848437
127	1	0	3.610521	-0.438895	7.500693
128	1	0	2.992962	0.187768	5.953347
129	1	0	7.027671	-1.386668	6.250562
130	1	0	5.963292	-1.319172	7.659770
131	1	0	6.628723	0.178254	6.994413
132	1	0	3.591532	-2.055686	4.841262
133	1	0	4.197677	-2.644150	6.409014
134	1	0	5.239679	-2.696487	4.972296
135	1	0	2.589711	7.815757	-4.501882
136	1	0	2.023780	7.067377	-6.001277
137	1	0	3.630660	6.673312	-5.380783
138	1	0	0.038073	5.250218	-3.399925
139	1	0	-0.068940	6.228602	-4.883442
140	1	0	0.445840	6.975448	-3.357251
141	1	0	3.036685	4.177652	-5.443535
142	1	0	1.449895	4.611717	-6.110695
143	1	0	1.559118	3.598275	-4.653589
144	1	0	-4.430003	0.810215	1.257057
145	1	0	-5.447088	-3.200410	0.109592
146	1	0	-7.081932	-2.344776	-1.472065
147	1	0	-6.039984	1.688036	-0.292049
148	1	0	-4.203924	8.329169	1.878603
149	1	0	-3.988610	6.999451	0.714937
150	1	0	-2.710356	7.390923	1.887090
151	1	0	-5.832999	7.875662	3.180761
152	1	0	-5.759595	6.546699	4.337446
153	1	0	-6.521031	6.291924	2.750153
154	1	0	-9.153814	-0.079600	-3.325796
155	1	0	-9.151496	-1.213843	-1.958969
156	1	0	-7.953014	-1.372544	-3.264344
157	1	0	-8.616570	1.894856	-2.776460
158	1	0	-6.983163	2.260630	-2.213172
159	1	0	-8.299057	2.049713	-1.035002

The total electronic energy was calculated to be –5078.0514908 Hartree.

Table S8. Optimized structure of **TS$_A$** (B3LYP-D3/6-31G(d))

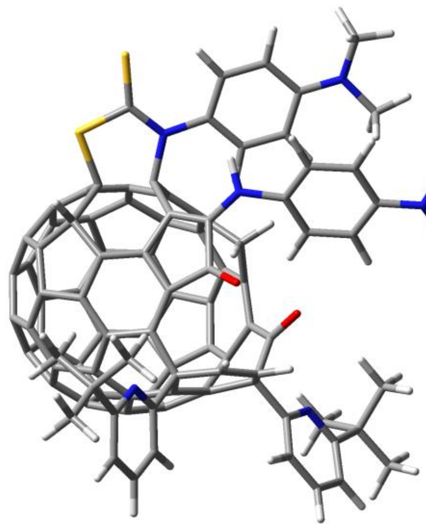

Standard orientation:

Center Number	Atomic Number	Atomic Type	Coordinates (Angstroms) X	Y	Z
1	8	0	-1.568457	1.273191	3.115981
2	8	0	-1.253785	3.111208	-0.324346
3	7	0	1.158032	5.109055	-1.441767
4	7	0	2.683633	0.815815	4.643392
5	7	0	-3.253471	-3.446787	0.188401
6	7	0	-3.622497	-0.413784	1.914838
7	6	0	-1.492450	-2.348478	-1.327301
8	6	0	-0.203851	-4.310843	-1.054060
9	6	0	3.857854	1.521284	-1.539527
10	6	0	3.765077	0.629755	0.748995
11	6	0	2.253423	2.425918	0.052459
12	6	0	2.663600	2.208587	-1.319703
13	6	0	1.082167	3.369793	0.265506
14	6	0	4.443824	0.741376	-0.470457
15	6	0	-1.474061	-1.105353	-1.963964
16	6	0	-0.223371	-3.315619	-3.277780
17	6	0	3.696391	-0.678670	1.395381
18	6	0	-0.797293	-3.433038	-2.000042
19	6	0	4.968819	-0.317849	-2.499770
20	6	0	-0.260215	-2.051642	-3.937462
21	6	0	2.437595	-0.979613	2.068350
22	6	0	-1.259590	0.313201	2.442544
23	6	0	2.747598	1.614195	1.050550
24	6	0	-0.761531	-4.088795	0.324978
25	6	0	-0.844231	-0.991344	-3.261088
26	6	0	3.004992	1.557580	3.585376
27	6	0	2.587088	6.871405	0.144157
28	6	0	0.586012	3.389151	1.686790
29	6	0	4.801612	-1.496370	-3.231860
30	6	0	-1.617714	-1.728939	1.240464
31	6	0	-0.093800	2.884351	-0.631138
32	6	0	0.627877	-2.619166	1.888595
33	6	0	0.400381	-4.006925	1.399540
34	6	0	2.001082	-2.310742	2.037796
35	6	0	2.030587	1.469907	2.397163

36	6	0	5.143473	-0.372940	-1.063139
37	6	0	4.895895	2.479002	4.714477
38	6	0	0.249182	2.093453	-1.828180
39	6	0	1.025460	-4.898802	-1.323035
40	6	0	2.088387	-4.906792	-0.294863
41	6	0	-1.668585	0.348585	-1.464255
42	6	0	-4.325350	0.813531	1.818593
43	6	0	1.037457	-3.941801	-3.585278
44	6	0	-2.286970	-0.643408	1.777922
45	6	0	-1.842279	-2.817593	0.155827
46	6	0	0.989491	2.535048	2.620743
47	6	0	1.513248	6.306876	-1.939745
48	6	0	-3.792001	1.954465	1.214451
49	6	0	4.157522	0.846278	-2.785132
50	6	0	1.673001	-4.685478	-2.592792
51	6	0	4.836160	-2.776745	-2.551473
52	6	0	5.039276	-2.831061	-1.168993
53	6	0	2.746214	-3.398352	1.477123
54	6	0	1.611391	2.101086	-2.333942
55	6	0	5.158615	-1.595798	-0.406964
56	6	0	1.493413	4.778268	-0.195347
57	6	0	-0.283305	0.328166	-3.404058
58	6	0	2.215235	5.629213	0.647419
59	6	0	-5.909840	3.149838	1.469924
60	6	0	-0.622555	1.112062	-2.275113
61	6	0	4.443340	-1.746757	0.843375
62	6	0	-4.561471	3.097857	1.048687
63	6	0	3.186624	0.793241	-3.775401
64	6	0	0.094493	-0.298915	2.200731
65	6	0	-5.658666	0.843882	2.235829
66	6	0	2.983474	-0.070335	6.879362
67	6	0	0.983117	-1.860922	-4.665983
68	6	0	1.438296	0.049917	2.310906
69	6	0	-0.260632	-1.618470	1.767910
70	6	0	0.905821	0.530877	-4.101428
71	6	0	1.895696	1.404041	-3.521128
72	6	0	3.965623	-3.118547	0.877429
73	6	0	1.077079	6.557377	-3.387487
74	6	0	1.812724	-4.350823	0.925706
75	6	0	4.570092	1.688476	5.818399
76	6	0	3.349810	-4.626559	-0.954949
77	6	0	3.444604	0.858311	5.751056
78	6	0	3.838348	-3.637147	-3.165942
79	6	0	2.235362	7.219537	-1.161890
80	6	0	3.178931	-2.878681	-4.217473
81	6	0	4.105200	2.420774	3.571649
82	6	0	1.566392	-0.593655	-4.730895
83	6	0	3.102929	-4.527391	-2.378355
84	6	0	-6.440451	1.980524	2.060090
85	6	0	1.802025	-3.030361	-4.421884
86	7	0	-6.681961	4.302153	1.307208
87	6	0	3.780599	-1.559407	-4.264097
88	6	0	2.989608	-0.434447	-4.528289
89	6	0	4.296794	-3.775664	-0.362910
90	6	0	1.530548	0.298946	7.254657
91	6	0	3.872757	0.036427	8.128411
92	6	0	3.020971	-1.522247	6.348551
93	6	0	1.453520	7.965136	-3.877387
94	6	0	-0.455674	6.380886	-3.476244
95	6	0	1.763789	5.504741	-4.288232
96	6	0	-4.419943	-2.663169	-0.191010
97	6	0	-4.267621	-1.354995	-0.664561
98	6	0	-5.753622	-3.105946	-0.082005
99	6	0	-6.827389	-2.295549	-0.436189
100	6	0	-5.327543	-0.538185	-1.021718
101	6	0	-6.659593	-0.985780	-0.930852
102	6	0	-6.187790	5.344138	0.421817
103	6	0	-8.121893	4.196890	1.467198
104	7	0	-7.729132	-0.179634	-1.284719
105	6	0	-9.068742	-0.576585	-0.884021
106	6	0	-7.485779	1.232196	-1.545090
107	6	0	-3.331813	-4.758896	0.578275
108	16	0	-4.605219	-5.799549	0.908901
109	16	0	-1.786062	-5.530306	0.809095
110	1	0	-4.204430	-1.219358	1.721038
111	1	0	3.148443	7.567923	0.761544
112	1	0	-0.197460	4.108195	1.900987
113	1	0	5.764766	3.131280	4.747879
114	1	0	-1.497210	0.456773	-0.395405
115	1	0	-2.680580	0.719005	-1.665847
116	1	0	0.115629	-4.659770	2.235036
117	1	0	0.541768	2.546222	3.607634
118	1	0	-2.771961	1.972373	0.858105
119	1	0	2.466627	5.319359	1.656622
120	1	0	-4.091327	3.945103	0.565202
121	1	0	-6.101248	-0.042643	2.683558
122	1	0	5.186716	1.724051	6.708612
123	1	0	2.522111	8.185915	-1.558551
124	1	0	4.331485	3.014265	2.691747
125	1	0	-7.472642	1.945387	2.386150
126	1	0	1.474944	1.320764	7.649109
127	1	0	1.154186	-0.384010	8.025506
128	1	0	0.878229	0.234574	6.379629
129	1	0	4.910463	-0.245805	7.914625
130	1	0	3.500820	-0.640320	8.905488
131	1	0	3.869259	1.051013	8.544219
132	1	0	2.404318	-1.619461	5.450512
133	1	0	2.645007	-2.214452	7.111386
134	1	0	4.045047	-1.820046	6.092836
135	1	0	0.987239	8.746424	-3.265456
136	1	0	1.107858	8.101400	-4.907935
137	1	0	2.538419	8.123227	-3.870993
138	1	0	-0.750775	5.388999	-3.123679
139	1	0	-0.788642	6.500486	-4.514128
140	1	0	-0.972930	7.129291	-2.863889
141	1	0	2.855084	5.605152	-4.249073
142	1	0	1.447279	5.635819	-5.329973
143	1	0	1.504466	4.491180	-3.970449
144	1	0	-3.302488	-0.905805	-0.677628
145	1	0	-5.975354	-4.088745	0.294269
146	1	0	-7.818864	-2.712242	-0.305271
147	1	0	-5.098905	0.480436	-1.308382
148	1	0	-6.904712	6.167883	0.402912
149	1	0	-6.033191	4.992461	-0.612332
150	1	0	-5.236589	5.746020	0.787326
151	1	0	-8.574666	5.176807	1.301431
152	1	0	-8.378988	3.886395	2.486218
153	1	0	-8.582104	3.478598	0.766426
154	1	0	-9.786060	0.160149	-1.251349
155	1	0	-9.179392	-0.655017	0.209796
156	1	0	-9.337625	-1.543716	-1.323649
157	1	0	-8.426447	1.709839	-1.828179
158	1	0	-6.790478	1.355670	-2.383622
159	1	0	-7.070955	1.757967	-0.672145

The total electronic energy was calculated to be –5078.0350732 Hartree.

An imaginary frequency was found at –67.37 cm^{-1}.

Table S9. Optimized structure of **TSB** (B3LYP-D3/6-31G(d))

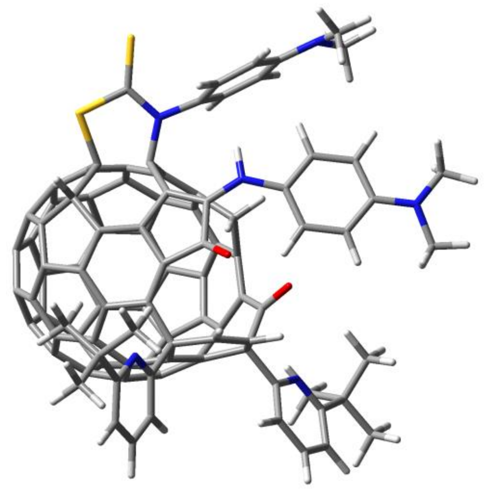

Standard orientation:

Center Number	Atomic Number	Atomic Type	X	Y	Z
1	8	0	-1.094962	1.318957	3.410646
2	8	0	-1.062609	3.236132	-0.165458
3	7	0	1.406347	4.957258	-1.514465
4	7	0	3.217874	0.648524	4.426226
5	7	0	-3.422163	-3.175601	0.639380
6	7	0	-3.283066	0.247175	1.639623
7	6	0	-1.827719	-2.228228	-1.032914
8	6	0	-0.692952	-4.297969	-0.881039
9	6	0	3.714041	1.151591	-1.890961
10	6	0	3.821703	0.272541	0.403042
11	6	0	2.381305	2.176915	-0.136842
12	6	0	2.608596	1.927326	-1.543857
13	6	0	1.331077	3.219039	0.194834
14	6	0	4.358442	0.330251	-0.889025
15	6	0	-1.831659	-0.998844	-1.688946
16	6	0	-0.901549	-3.319325	-3.102647
17	6	0	3.732962	-1.024315	1.071878
18	6	0	-1.317338	-3.375943	-1.764041
19	6	0	4.563482	-0.764852	-2.956055
20	6	0	-0.920795	-2.059560	-3.773807
21	6	0	2.556877	-1.225363	1.912233
22	6	0	-0.927474	0.419476	2.613002
23	6	0	2.928837	1.336025	0.808433
24	6	0	-1.061774	-4.027426	0.551722
25	6	0	-1.347918	-0.949954	-3.057145
26	6	0	3.494080	1.298401	3.296781
27	6	0	3.141761	6.565376	-0.077439
28	6	0	0.999056	3.274863	1.661738
29	6	0	4.220409	-1.925920	-3.653516
30	6	0	-1.591369	-1.567519	1.490801
31	6	0	0.022528	2.862566	-0.575491
32	6	0	0.597660	-2.714099	1.989238
33	6	0	0.205174	-4.069299	1.498211
34	6	0	2.002513	-2.514708	1.946330
35	6	0	2.379706	1.261343	2.235344
36	6	0	4.897718	-0.833865	-1.548443
37	6	0	5.594933	2.040489	4.154068
38	6	0	0.148175	2.029196	-1.788096
39	6	0	0.433904	-4.997736	-1.284961
40	6	0	1.601963	-5.095444	-0.383809
41	6	0	-1.873838	0.466672	-1.201718
42	6	0	-3.777344	1.579618	1.565937
43	6	0	0.257320	-4.051422	-3.545937
44	6	0	-2.059305	-0.308663	1.849591
45	6	0	-2.013653	-2.655825	0.473184
46	6	0	1.448585	2.401899	2.557526
47	6	0	1.823735	6.119629	-2.046395
48	6	0	-5.101219	1.710580	1.119384
49	6	0	3.818336	0.458905	-3.157540
50	6	0	0.941321	-4.844071	-2.624500
51	6	0	4.230215	-3.203758	-2.967693
52	6	0	4.586847	-3.273664	-1.616969
53	6	0	2.582015	-3.651102	1.290287
54	6	0	1.443906	1.912585	-2.433951
55	6	0	4.893235	-2.052850	-0.885880
56	6	0	1.826653	4.587209	-0.305724
57	6	0	-0.704333	0.320603	-3.281788
58	6	0	2.704919	5.359579	0.461066
59	6	0	-5.016742	4.141728	1.248456
60	6	0	-0.861972	1.140162	-2.135216
61	6	0	4.322162	-2.145945	0.442500
62	6	0	-5.710654	2.947300	0.961066
63	6	0	2.739892	0.485111	-4.030654
64	6	0	0.321849	-0.354703	2.328434
65	6	0	-3.076174	2.759360	1.837052
66	6	0	3.672381	-0.159858	6.664843
67	6	0	0.243797	-1.976855	-4.640044
68	6	0	1.687047	-0.115798	2.266918
69	6	0	-0.215740	-1.637293	1.974831
70	6	0	0.420699	0.414186	-4.101168
71	6	0	1.538307	1.198205	-3.640127
72	6	0	3.739643	-3.471833	0.545214
73	6	0	1.277392	6.418691	-3.446731
74	6	0	1.517339	-4.519206	0.857280
75	6	0	5.314829	1.343727	5.330915
76	6	0	2.794689	-4.923069	-1.189467
77	6	0	4.102383	0.651244	5.437895
78	6	0	3.101896	-3.980476	-3.454739
79	6	0	2.699542	6.955436	-1.343446
80	6	0	2.389027	-3.171866	-4.432315
81	6	0	4.673805	2.025584	3.111295
82	6	0	0.915567	-0.762343	-4.785925
83	6	0	2.393511	-4.805388	-2.577163
84	6	0	-3.681055	4.000195	1.677058
85	6	0	0.989858	-3.208896	-4.476217
86	7	0	-5.619019	5.393657	1.115334
87	6	0	3.085395	-1.906093	-4.561192
88	6	0	2.361384	-0.721453	-4.745367
89	6	0	3.869631	-4.153050	-0.719345
90	6	0	3.466554	-1.626948	6.221626
91	6	0	2.331743	0.409570	7.181429
92	6	0	4.713067	-0.111953	7.795173
93	6	0	1.737779	7.786814	-3.975657
94	6	0	-0.266665	6.389045	-3.387358
95	6	0	1.773588	5.310799	-4.404541

96	6	0	-4.586807	-2.354897	0.449679
97	6	0	-5.423453	-2.108621	1.546597
98	6	0	-5.008671	-1.946740	-0.818766
99	6	0	-6.198045	-1.245530	-0.979554
100	6	0	-6.623850	-1.432937	1.394062
101	6	0	-7.041990	-0.967669	0.122239
102	6	0	-6.878327	5.479158	0.397179
103	6	0	-4.766264	6.571905	1.102854
104	7	0	-8.208377	-0.237468	-0.026740
105	6	0	-8.456303	0.444162	-1.288725
106	6	0	-8.880471	0.268668	1.162696
107	6	0	-3.649110	-4.501034	0.892473
108	16	0	-5.108215	-5.288780	1.020935
109	16	0	-2.169063	-5.393811	1.128610
110	1	0	-3.949558	-0.417423	1.262603
111	1	0	3.824002	7.200691	0.481521
112	1	0	0.307631	4.057938	1.955476
113	1	0	6.529282	2.586524	4.052372
114	1	0	-1.561296	0.583276	-0.170669
115	1	0	-2.884039	0.888974	-1.270246
116	1	0	-0.042315	-4.722630	2.345299
117	1	0	1.123948	2.454810	3.590394
118	1	0	-5.666029	0.823418	0.871190
119	1	0	3.025140	5.018762	1.440400
120	1	0	-6.731075	2.966768	0.596120
121	1	0	-2.047191	2.738994	2.148464
122	1	0	6.031562	1.344775	6.143435
123	1	0	3.036016	7.893539	-1.767918
124	1	0	4.861874	2.547643	2.178875
125	1	0	-3.070339	4.872795	1.872235
126	1	0	2.736015	-1.684398	5.409814
127	1	0	3.106923	-2.229667	7.064157
128	1	0	4.406847	-2.065054	5.865731
129	1	0	2.450380	1.445985	7.519932
130	1	0	1.969807	-0.185510	8.028302
131	1	0	1.576123	0.391977	6.391279
132	1	0	5.676551	-0.529815	7.480298
133	1	0	4.359830	-0.703389	8.646990
134	1	0	4.880463	0.911895	8.150397
135	1	0	1.406476	8.605979	-3.326382
136	1	0	1.311974	7.959630	-4.970127
137	1	0	2.828688	7.841383	-4.070931
138	1	0	-0.620215	5.428574	-3.003083
139	1	0	-0.685524	6.545313	-4.388633
140	1	0	-0.648974	7.180320	-2.731202
141	1	0	2.868389	5.308966	-4.468983
142	1	0	1.374024	5.476312	-5.412224
143	1	0	1.452175	4.324367	-4.059500
144	1	0	-5.119650	-2.455555	2.528382
145	1	0	-4.407225	-2.181079	-1.688111
146	1	0	-6.469139	-0.921120	-1.975978
147	1	0	-7.224386	-1.248188	2.274959
148	1	0	-7.214982	6.518139	0.383449
149	1	0	-7.653829	4.891679	0.902625
150	1	0	-6.806405	5.123275	-0.644977
151	1	0	-5.386996	7.461944	0.977792
152	1	0	-4.017286	6.550787	0.294183
153	1	0	-4.234426	6.675343	2.055227
154	1	0	-9.408946	0.973138	-1.228312
155	1	0	-8.531240	-0.273112	-2.113624
156	1	0	-7.667248	1.172864	-1.535019
157	1	0	-9.768254	0.827011	0.860607
158	1	0	-8.234941	0.933813	1.757811
159	1	0	-9.214043	-0.554027	1.804582

The total electronic energy was calculated to be –5078.0558252 Hartree.

An imaginary frequency was found at –37.91 cm^{-1}.

6. IR Spectra

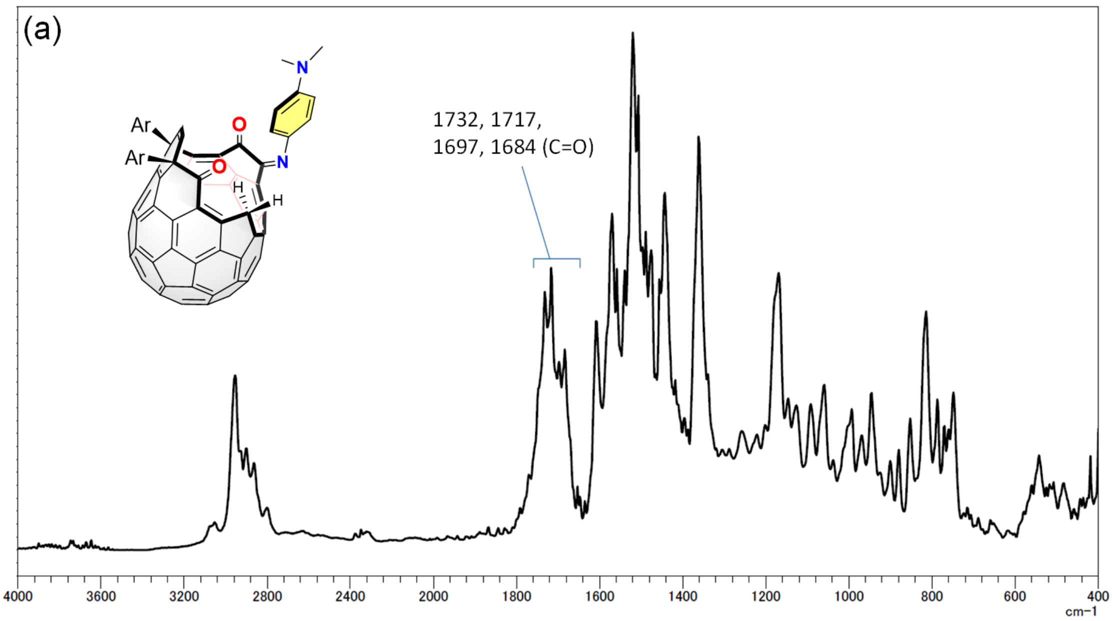

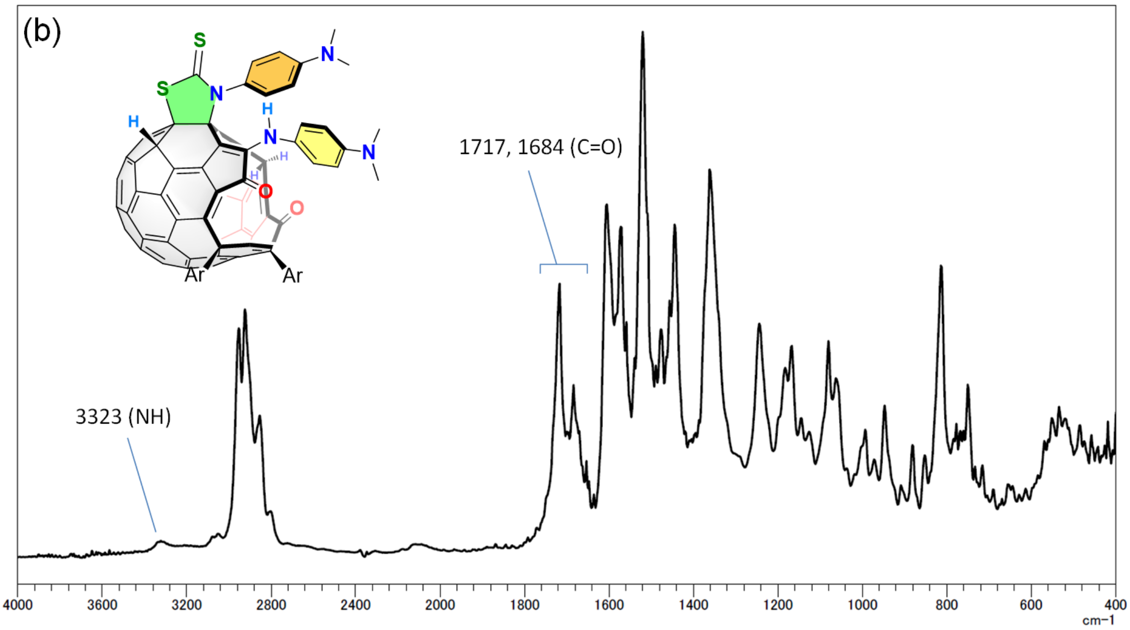

Figure S12. IR spectra (KBr) of (a) **2** and (b) **3**.

8. Structural Isomer 3’

The structural isomer **3’** was calculated to be less stable by ΔE +1.4 kcal/mol than **3** (B3LYP-D3/6-31G(d)).

Table S10. Optimized structure of **3’** (B3LYP-D3/6-31G(d))

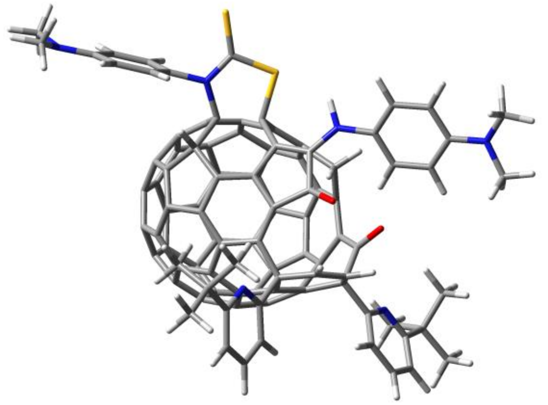

Standard orientation:

Center Number	Atomic Number	Atomic Type	Coordinates (Angstroms) X	Y	Z
1	8	0	1.744706	3.251164	1.717934
2	8	0	3.903541	1.546572	-0.980144
3	7	0	5.885635	-1.116730	-0.672963
4	7	0	1.033535	0.030342	5.041434
5	7	0	-4.559146	2.093820	-0.958241
6	7	0	0.297388	4.224905	-0.752755
7	6	0	-1.473991	1.103113	-2.426325
8	6	0	-3.493331	0.031370	-1.815945
9	6	0	2.318653	-3.608558	0.054974
10	6	0	1.211080	-2.566215	1.987209
11	6	0	3.069865	-1.440815	0.857653
12	6	0	2.981626	-2.410903	-0.211710
13	6	0	4.001317	-0.266493	0.622063
14	6	0	1.436746	-3.700705	1.198293
15	6	0	-0.189453	0.908381	-2.921619
16	6	0	-2.281133	-0.886723	-3.725748
17	6	0	-0.153227	-2.272523	2.425560
18	6	0	-2.509727	0.170155	-2.833274
19	6	0	0.581303	-5.099221	-0.483518
20	6	0	-0.959714	-1.090644	-4.228207
21	6	0	-0.512737	-0.859774	2.477233
22	6	0	0.842481	2.628950	1.201154
23	6	0	2.170708	-1.486952	1.901398
24	6	0	-3.402056	1.155145	-0.801567
25	6	0	0.037454	-0.224943	-3.799839
26	6	0	1.899669	-0.643401	4.287692
27	6	0	7.491309	-1.502345	1.547919
28	6	0	3.878973	0.804035	1.672862
29	6	0	-0.517936	-5.313326	-1.319197
30	6	0	-1.081772	2.292090	-0.144800
31	6	0	3.641792	0.376712	-0.751971
32	6	0	-2.117008	0.642221	1.385928
33	6	0	-3.435383	0.575601	0.678563
34	6	0	-1.834279	-0.526693	2.135359
35	6	0	1.911815	-0.271130	2.794437
36	6	0	0.383700	-4.631528	0.872785
37	6	0	2.747080	-1.819614	6.185015
38	6	0	2.946713	-0.467308	-1.743226
39	6	0	-4.056408	-1.211847	-1.567564
40	6	0	-4.179042	-1.716238	-0.181791
41	6	0	1.206108	1.370929	-2.447512
42	6	0	1.483074	4.993764	-0.863392
43	6	0	-2.885649	-2.174109	-3.502216
44	6	0	0.009809	3.120916	-0.010382
45	6	0	-2.047875	1.982061	-1.274637
46	6	0	2.950345	0.806436	2.623248
47	6	0	7.117825	-1.631498	-0.830259
48	6	0	2.760732	4.434850	-0.794392
49	6	0	1.770871	-4.448876	-0.988695
50	6	0	-3.729660	-2.339383	-2.403939
51	6	0	-1.858611	-5.094973	-0.810885
52	6	0	-2.049564	-4.676340	0.510066
53	6	0	-2.859476	-1.482736	1.834156
54	6	0	2.983482	-1.911793	-1.589399
55	6	0	-0.896662	-4.405388	1.357176
56	6	0	5.444850	-0.797226	0.543162
57	6	0	1.360769	-0.742890	-3.557605
58	6	0	6.213315	-0.973333	1.698028
59	6	0	3.793178	6.607810	-1.238108
60	6	0	2.036715	0.093881	-2.631209
61	6	0	-1.165566	-3.225974	2.154022
62	6	0	3.889161	5.224795	-0.962587
63	6	0	1.822537	-4.010139	-2.304427
64	6	0	0.189449	1.330024	1.643406
65	6	0	1.366725	6.362381	-1.126860
66	6	0	-0.098052	0.610054	7.104782
67	6	0	-0.711699	-2.519856	-4.310864
68	6	0	0.506252	0.180716	2.356206
69	6	0	-1.085563	1.412861	0.998261
70	6	0	1.612664	-2.113199	-3.626830
71	6	0	2.411054	-2.715011	-2.588868
72	6	0	-2.528848	-2.830826	1.848670
73	6	0	7.494804	-1.973696	-2.275727
74	6	0	-3.738049	-0.915072	0.838956
75	6	0	1.825238	-1.127347	6.973611
76	6	0	-3.844881	-3.127890	-0.197858
77	6	0	0.973347	-0.198332	6.365031
78	6	0	-2.648348	-4.496850	-1.874761
79	6	0	7.954161	-1.836721	0.273841

80	6	0	-1.785169	-4.334915	-3.035768
81	6	0	2.793619	-1.580941	4.815602
82	6	0	0.551520	-3.025646	-3.999939
83	6	0	-3.604490	-3.523898	-1.571127
84	6	0	2.493663	7.156402	-1.311324
85	6	0	-1.904967	-3.193860	-3.837562
86	7	0	4.928918	7.389577	-1.446207
87	6	0	-0.471972	-4.848301	-2.694960
88	6	0	0.677679	-4.208957	-3.176312
89	6	0	-3.062468	-3.691924	0.821890
90	6	0	-1.476667	0.213090	6.526980
91	6	0	0.150794	2.112433	6.843413
92	6	0	-0.088956	0.350482	8.619836
93	6	0	8.941271	-2.480113	-2.399167
94	6	0	7.323035	-0.706894	-3.143678
95	6	0	6.528571	-3.071390	-2.778735
96	6	0	-5.876666	1.665288	-0.572263
97	6	0	-6.634488	0.839557	-1.404212
98	6	0	-6.429058	2.099477	0.634684
99	6	0	-7.693178	1.684273	1.029643
100	6	0	-7.899940	0.415810	-1.019243
101	6	0	-8.459263	0.811516	0.219167
102	6	0	6.226215	6.841571	-1.086365
103	6	0	4.787909	8.835370	-1.451839
104	7	0	-9.706401	0.365290	0.621633
105	6	0	-10.331554	0.954456	1.794435
106	6	0	-10.541404	-0.365332	-0.317659
107	6	0	-4.395718	3.277683	-1.602730
108	16	0	-5.553549	4.403310	-1.978182
109	16	0	-2.711848	3.539077	-2.065936
110	1	0	-0.495795	4.601600	-1.258690
111	1	0	8.126324	-1.656385	2.416592
112	1	0	4.567638	1.636619	1.576284
113	1	0	3.418340	-2.543911	6.639215
114	1	0	1.220655	1.692425	-1.411047
115	1	0	1.577247	2.214085	-3.043351
116	1	0	-4.184197	1.161564	1.220768
117	1	0	2.870625	1.641190	3.310214
118	1	0	2.894181	3.380289	-0.606158
119	1	0	5.816130	-0.701504	2.670541
120	1	0	4.853172	4.736167	-0.893169
121	1	0	0.381208	6.820728	-1.178206
122	1	0	1.776829	-1.315049	8.039579
123	1	0	8.948345	-2.249052	0.151180
124	1	0	3.488859	-2.105873	4.168321
125	1	0	2.348099	8.211821	-1.506365
126	1	0	-1.502830	0.376337	5.445735
127	1	0	-2.269098	0.810574	6.993653
128	1	0	-1.691302	-0.845581	6.716849
129	1	0	1.117326	2.427667	7.254634
130	1	0	-0.633404	2.713739	7.318632
131	1	0	0.151930	2.320601	5.770145
132	1	0	-0.297984	-0.699794	8.855040
133	1	0	-0.863455	0.956124	9.103206
134	1	0	0.872896	0.620949	9.071603
135	1	0	9.664777	-1.733324	-2.051065
136	1	0	9.167238	-2.696061	-3.449059
137	1	0	9.099481	-3.404908	-1.831897
138	1	0	6.302963	-0.321242	-3.067614
139	1	0	7.534645	-0.938534	-4.194296
140	1	0	8.011423	0.084496	-2.823795
141	1	0	6.628623	-3.986674	-2.182971
142	1	0	6.751060	-3.319299	-3.823505
143	1	0	5.490067	-2.735519	-2.715223
144	1	0	-6.234903	0.535058	-2.364627
145	1	0	-5.869520	2.790597	1.258443
146	1	0	-8.085631	2.052847	1.968904
147	1	0	-8.456197	-0.219066	-1.697185
148	1	0	7.000043	7.581694	-1.301397
149	1	0	6.451900	5.949484	-1.681835
150	1	0	6.294671	6.565488	-0.021093
151	1	0	5.766429	9.291088	-1.617692
152	1	0	4.375897	9.230929	-0.508364
153	1	0	4.133572	9.160455	-2.269379
154	1	0	-11.299601	0.479015	1.962154
155	1	0	-10.492844	2.038636	1.688351
156	1	0	-9.722410	0.786482	2.690972
157	1	0	-11.472994	-0.648877	0.175453
158	1	0	-10.047597	-1.288062	-0.645543
159	1	0	-10.790169	0.226670	-1.212331

The total electronic energy was calculated to be –5078.0624737 Hartree.

8. References

(1) (*a*) K. Kurotobi and Y. Murata, *Science*, 2011, **333**, 613–616; (*b*) Y. Hashikawa, M. Murata, A. Wakamiya and Y. Murata, *J. Am. Chem. Soc.*, 2017, **139**, 16350−16358.
